# GLS1‐RNA Polymerase II Axis Mediates Glutamine‐Dependent Hepatoprotective Effects on Alcoholic Liver Disease in High‐Protein Diets

**DOI:** 10.1002/advs.202502738

**Published:** 2025-09-11

**Authors:** Wenbiao Wu, Haowen Jiang, Yichang Liu, Chang Peng, Hanlin Wang, Wenhua Yang, Zan Lyu, Yan Sun, Huan Ma, Hongyu Gu, Weijuan Kan, Liya Jing, Tiancheng Dong, Chunmei Xia, Saifei Lei, Rui Wu, Jinlong Li, Jia Li

**Affiliations:** ^1^ Hangzhou Institute for Advanced Study University of Chinese Academy of Sciences Hangzhou 310000 China; ^2^ University of Chinese Academy of Sciences No.19A Yuquan Road Beijing 100049 China; ^3^ State Key Laboratory of Chemical Biology Shanghai Institute of Materia Medica Chinese Academy of Sciences Shanghai 201203 China; ^4^ School of pharmacy Nantong University Nantong 226019 China; ^5^ School of Life Science and Technology Shanghai Tech University Shanghai 201210 China; ^6^ College of Pharmacy Fudan University Shanghai 210023 China; ^7^ Lingang laboratory Shanghai 201203 China; ^8^ Zhongshan Institute for Drug Discovery Shanghai Institute of Materia Medica Chinese Academy of Sciences Zhongshan Tsuihang New District Guangdong 528400 China; ^9^ Shandong Laboratory of Yantai Drug Discovery Bohai Rim Advanced Research Institute for Drug Discovery Yantai Shandong 264117 China

**Keywords:** alcoholic fatty liver disease, GLS1, hepatic lipogenesis, high‐protein diets, RNA polymerase II

## Abstract

The RNA polymerase II (RNA pol II) complex is essential for gene transcription throughout life, and numerous cofactors have been identified as critical for diverse transcriptional processes. Herein, it is discovered that the RNA pol II complex is modulated by glutaminase 1 (GLS1), which affects lipid metabolism. In alcoholic fatty liver disease (AFLD), RNA pol II activation is observed, whereas RNA pol II inhibition reverse hepatic steatosis. Furthermore, high‐protein diets are recognized for their adjuvant effect on patients with AFLD; glutamine is indispensable for its protective effects against hepatic steatosis, which is dependent on RNA pol II. Mechanistically, GLS1 acts as a chaperone that affects the RNA pol II complex in the nucleus by interacting with its subunits, POLR2H and POLR2E. In vivo studies have shown that hepatic overexpression of GLS1 ameliorates alcohol‐induced fatty liver, whereas deficiency worsens this condition. Moreover, the overexpression of POLR2E or POLR2H, but not the truncated variants, abolishes the protective effects of GLS1 against alcohol‐induced fatty liver. Thus, the study clarifies GLS1 as a cofactor involved in assembling the RNA pol II complex, regulating hepatic steatosis, and provides foundational insights for future therapeutic approaches in AFLD.

## Graphical abstract

GLS1 interacts with RNA polymerase II subunits, ROLR2H and POLR2E, resulting in significant suppression of RNA polymerase II activity. Importantly, the investigation demonstrates that high‐protein diets, particularly glutamine, exert protective effects against alcoholic fatty liver through GLS1‐RNA polymerase II axis. Thus, GLS1‐RNA pol II axis is essential for alcohol‐induced fatty liver disease, indicating its potential as a novel therapeutic target.

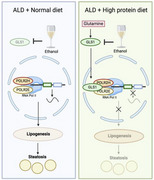

## 1 Introduction

Alcoholic fatty liver disease (AFLD) is highly prevalent worldwide and is increasingly recognized as a significant cause of morbidity and mortality.^[^ ^1^ ^]^ AFLD typically progresses from alcoholic fatty liver (AFL) to alcoholic steatohepatitis (ASH), which is characterized by hepatic inflammation. Chronic ASH can progress to fibrosis and cirrhosis and, in severe cases, hepatocellular cancer (HCC).^[^ ^2^ ^]^ Alcohol abstinence is crucial for preventing the onset and development of AFLD. However, patients with severe liver complications stemming from AFLD or alcohol addiction often require pharmacological intervention. Despite the development of several drugs aimed at achieving sustained abstinence from alcohol, the remission rates of AFLD and associated diseases remain limited.^[^ ^3^, ^4^, ^5^ ^]^ Therefore, there is an urgent need to develop effective therapies that can slow the progression of liver disease and reduce mortality rates.

Fatty liver, recognized as hepatic steatosis characterized by the deposition of fat in hepatocytes, is the earliest response to heavy drinking.^[^ ^1^ ^]^ Without prompt intervention at the stage of steatosis, it can progress to more severe conditions, such as fibrosis and cirrhosis.^[^ ^6^ ^]^ Although it is similar to hepatic steatosis caused by metabolic dysfunction associated with fatty liver disease (MAFLD), there are currently no lipid‐lowering medications used for the treatment of AFLD, indicating that the pathogenesis of alcohol‐related steatosis is different from that of MAFLD. Alcohol induces hepatic steatosis through multiple pathways.^[^ ^7^ ^]^ Previous researches have shown that alcohol intake upregulates hepatic CD36 expression, increasing fatty acid uptake and thereby promoting the accumulation of triglycerides (TGs).^[^ ^8^ ^]^ Concurrently, alcohol impedes the secretion of cytoplasmic lipoproteins in hepatocytes, leading to the accumulation of TGs. Moreover, alcohol activates key transcription factors to promote the transcription and expression of key downstream lipid synthesis genes, such as sterol regulatory element‐binding protein (SREBP1c),^[^ ^9^ ^]^ carbohydrate response element‐binding protein (ChREBP),^[^ ^10^ ^]^ and peroxisome proliferator‐activated receptor‐γ (PPARγ), thereby exacerbating hepatic lipid accumulation.^[^ ^11^, ^12^ ^]^ Additionally, alcohol can induce mitochondrial damage, primarily affecting mitochondrial stability, autophagy, and oxidative stress, leading to a decline in mitochondrial lipid oxidation and decomposition, resulting in hepatic lipid accumulation.^[^ ^13^, ^14^ ^]^ Alcohol can also downregulate the expression of peroxisome proliferator‐activated receptor‐alpha (PPARα), thereby inhibiting mitochondrial function and oxidative decomposition and promoting lipid accumulation in liver cells.^[^ ^15^, ^16^ ^]^ However, in animal models, the recovery of mitochondrial oxidative capacity by genetic manipulation does not significantly improve alcohol‐induced fatty liver or the progression of AFLD,^[^ ^7^ ^]^ suggesting that other pathogenic factors are involved in alcohol‐induced hepatic steatosis. Therefore, the underlying mechanism of alcohol‐induced fatty liver remains to be elucidated.

Several clinical studies have highlighted that protein energy malnutrition is present in almost every patient with AFLD and is associated with a poor prognosis.^[^ ^17^ ^]^ The European Society for Clinical Nutrition and Metabolism (ESPEN) and the Chinese Medical Association have recommendations for daily energy intake and daily protein intake for AFLD patients.^[^ ^18^, ^19^ ^]^ Glutamine, one of the most abundant free amino acids in the human body, reportedly has beneficial effects on ethanol‐induced liver injury and protein synthesis in skeletal muscle.^[^ ^20^ ^]^ Overall, it is well accepted that protein is beneficial to patients with AFLD. However, many questions remain unanswered regarding the associations among protein energy malnutrition (especially indispensable amino acids), glutamine, and ethanol‐induced hepatic steatosis.

RNA polymerase II (RNA pol II) is a 12‐subunit enzyme complex responsible for all protein‐coding genes and many noncoding RNAs in eukaryotic genomes. Precise spatiotemporally regulated transcription by the RNA pol II complex is the net outcome of intricate choreography among numerous elements,^[^ ^21^ ^]^ such as a set of general transcription factors and TATA‐binding proteins (TBPs).^[^ ^22^, ^23^ ^]^ Previous studies have shown that the RNA pol II complex plays an essential role in the regulation of lipid metabolism.^[^ ^24^, ^25^ ^]^ It has been reported that RNA pol II is involved in SREBP‐dependent transcription and splicing of lipogenesis‐related enzymes.^[^ ^26^ ^]^ Additionally, RNA pol II is crucial for the efficient splicing of genes involved in mitochondrial biogenesis, which contributes to hepatic lipogenesis.^[^ ^27^ ^]^ Despite these roles in the regulation of lipid metabolism, the regulatory effects of the RNA pol II complex on the pathology of alcohol‐induced fatty liver are unclear.

Here, we elucidated a regulatory model of the role of the RNA pol II complex in hepatic steatosis during the development of AFLD. We observed an increase in RNA pol II complex activity in the liver during AFLD, and blockade of RNA pol II activity by α‐amanitin prevented hepatic steatosis in AFLD, indicating the regulatory involvement of RNA pol II in AFLD. A high‐protein diet was shown to be an effective strategy to ameliorate hepatic steatosis in AFLD, affecting RNA pol II activity. Moreover, glutamine, but not glutamate, was identified as an indispensable amino acid in the high‐protein diet for manipulating hepatic steatosis in AFLD. Moreover, we found that the effects of glutamine on hepatic steatosis depended on glutaminase 1 (GLS1). Mechanistically, glutamine was found to stabilize GLS1, thereby promoting its interaction with RNA polymerase subunit H (POLR2H) and RNA polymerase subunit E (POLR2E), which reduced the activity of RNA pol II, affecting hepatic steatosis in AFLD. Together, these results highlight the regulatory role of RNA pol II activity by GLS1 in hepatic lipid metabolism and provide foundational insights for future therapeutic approaches in AFLD.

## 2 Results

### 2.1 The RNA pol II Transcriptional Pathway is Involved in the Process of AFLD

**Figure 1:**
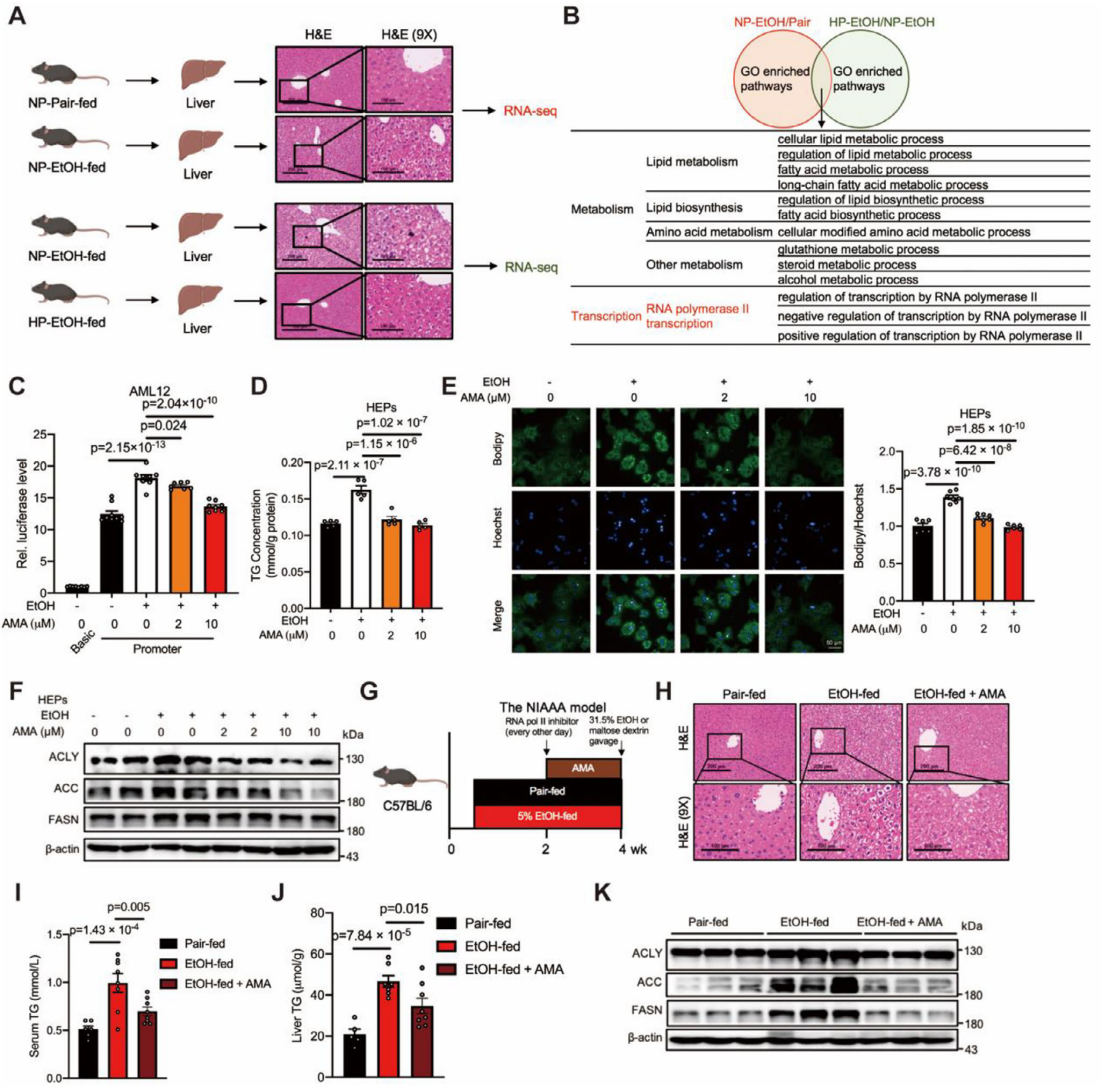
The RNA pol II transcriptional pathway is involved in the process of alcoholic hepatic steatosis. A) Schematic illustrating the groups and procedures of the normal‐protein diet and high‐protein diet AFLD model. n = 6–8 biologically independent mice per group. B) The strategy for identification of candidate pathways for alcohol‐induced lipogenesis in liver. Venn diagram showing the common pathways identified at the overlap of alcohol‐fed/vehicle‐fed groups’ and high‐protein‐diet/normal‐protein‐diet AFLD groups’ transcriptome data. C) RNA pol II activity of AML12 cells expressing pGL3‐Basic or pGL3‐Promoter treated with RNA pol II inhibitor (α‐Amanitin, AMA; 2, 10 µm) for 12 h. Cells were serum starved overnight followed by ethanol (400 mm) stimulation for 1 h. n = 8‐9. D) TG levels of the primary hepatocytes of C57BL/6J mice treated with α‐Amanitin (2, 10 µm) and ethanol (600 mm) for 24 h. The amount was normalized to the protein content. n = 5. E) Bodipy levels of the primary hepatocytes described in D). Scale bars, 50 µm. The amount was normalized to the hoechst content. n = 6‐7. F) Western blots of ACLY, ACC, FASN levels in the primary hepatocytes described in (D); β‐actin serves as a loading control. G) Schematic illustrating the groups and procedures of the mouse model. C57BL/6J mice were injected with vehicle or α‐Amanitin (0.15 mg kg^−1^ body weight, i.p., every other day) for the last 2 weeks of ethanol feeding. n = 6–8 biologically independent mice per group. H) Representative H&E staining of liver sections in the indicated group in (G). Scale bars, 100 and 200 µm. I,J) Serum TG and liver TG levels in the indicated group in (G); n = 5–8. K) Western blots of ACLY, ACC, FASN levels in liver of the mice described in (G); β‐actin served as the loading control. Data in (C‐E), (I‐J) are presented as the mean ± SEM, determined by one‐way ANOVA and Fisher's LSD test.

To explore the mechanisms by which alcohol affects hepatic steatosis under conditions of a high‐protein diet, we established an AFLD mouse model by administering mice 5% ethanol for 4 weeks while feeding them either a normal or high‐protein diet, as previously described^[^ ^28^ ^]^ (**Figure** **1**A). The results showed that alcohol‐induced hepatic steatosis could be reduced in mice fed a high‐protein diet without affecting body weight or food intake (Figure S1A–G, Supporting Information). Transcriptomic analyses of liver samples from both alcohol‐fed and control mice, as well as from mice fed a high‐protein diet versus a normal diet, were then conducted (Figures S1H–K and S2A–D, Supporting Information). Gene Ontology (GO) analyses revealed that the differentially expressed genes (DEGs) enriched in both the alcohol‐fed group versus the control group and the high‐protein diet group versus the normal diet group were involved in several metabolic pathways, including lipid metabolism and amino acid metabolism. These findings indicated that both lipid and amino acid metabolism were affected by alcohol and partially reversed by high protein supplementation. Surprisingly, we found that RNA pol II transcriptional pathways were induced by alcohol and inhibited by high‐protein supplementation (Figure 1B).

We subsequently reanalyzed the lipid metabolism pathway and RNA pol II transcriptional pathways based on a previously published study in AFLD patients and murine models of AFLD, and GO analysis revealed that both pathways were induced in AFLD patients (Figure S2E, Supporting Information) and AFLD murine models (Figure S2F,G, Supporting Information). Previous studies have reported that RNA pol II plays a critical role in SREBP‐dependent transcription,^[^ ^26^ ^]^ the splicing of lipogenesis‐related enzymes and mitochondrial biogenesis‐related genes,^[^ ^27^ ^]^ thereby affecting hepatic lipid metabolism. Thus, we hypothesized that RNA pol II might participate in the process of liver lipid deposition in AFLD.

We subsequently established an AML12 cell line expressing the pGL3‐SV40 promoter, which is closely associated with RNA pol II activity^[^ ^29^ ^]^ (Figure S2H, Supporting Information). Our results revealed that alcohol increased RNA pol II activity (Figure S2I, Supporting Information). Additionally, we found that alcohol increased the intracellular TG content in hepatocytes (Figure S2J, Supporting Information). To further elucidate the role of RNA pol II in hepatic steatosis, we examined the effects of RNA pol II inhibition on hepatocytes. Treatment with an RNA pol II inhibitor (α‐amanitin; AMA) significantly decreased RNA pol II activity (Figure 1C; Figure S3A, Supporting Information). The intracellular TG content was decreased by AMA treatment (Figure 1D,E). Furthermore, the expression of lipogenic genes and proteins was decreased by AMA treatment in cells (Figure 1F; Figure S3B, Supporting Information). To assess the physiological function of RNA pol II in vivo, we created a mouse model by the intraperitoneal injection of AMA every other day for the last 2 weeks of alcohol consumption (Figure 1G). The food intake was kept constant, and the body weight curves remained similar (Figure S3C,D, Supporting Information). A reduction in the serum TG concentration was observed in the AMA‐treated mice (Figure 1I). In the liver, compared with the alcohol‐fed group, the AMA‐treated group presented a reduction in liver lipid deposition, as evidenced by hematoxylin‐eosin (H&E) staining (Figure 1H) and a reduction in the hepatic TG content (Figure 1J). Additionally, the expression of lipogenic genes and proteins was decreased by AMA (Figure 1K; Figure S3E, Supporting Information). Taken together, these data suggest that the inhibition of RNA pol II activity mitigates alcohol‐induced fatty liver.

### 2.2 Glutamine Protects Against Alcohol‐Induced Hepatic Steatosis Through the Inhibition of RNA pol II Activity

**Figure 2:**
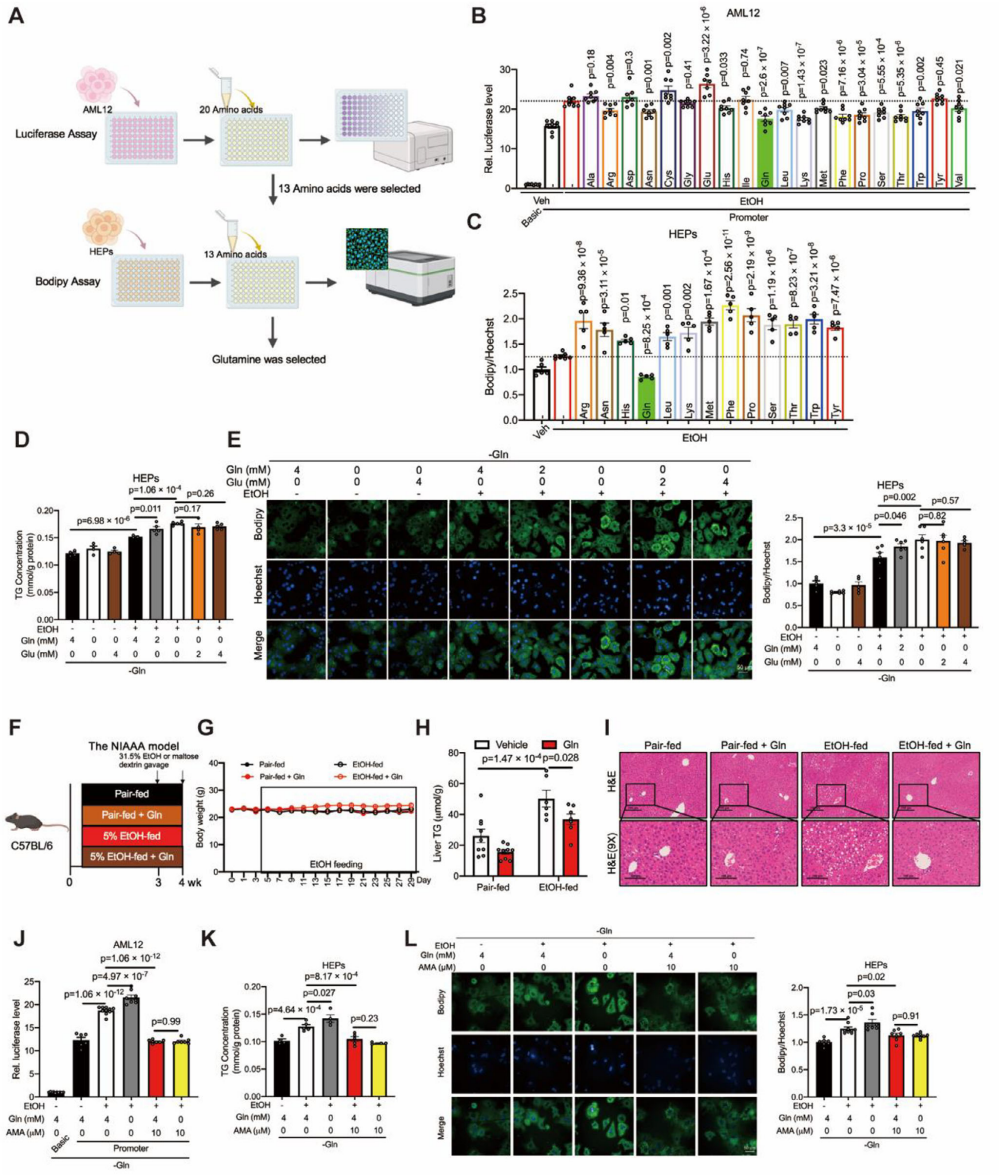
Glutamine instead of glutamate reduces triacylglycerol deposition through decreasing RNA pol II activity. A) Schematic diagram illustrating the screening strategy of amino acids. B) RNA pol II activity of AML12 cells expressing pGL3‐Basic or pGL3‐Promoter treated with 20 amino acids (4 mm) for 12 h. Cells were serum starved overnight followed by ethanol (400 mm) stimulation for 1 h. n = 7‐8. C) Bodipy levels of the primary hepatocytes treated with 13 amino acids (4 mm) and ethanol (600 mm) for 24 h. The amount was normalized to the hoechst content. n = 5–7. D) TG levels of the primary hepatocytes treated with glutamine (2 or 4 mm) or glutamate (2 or 4 mm) and ethanol (600 mm) in glutamine‐free medium for 24 h. The amount was normalized to the protein content. n = 3–5. E) Bodipy levels of the primary hepatocytes described in (D); Scale bars, 50 µm. The amount was normalized to the hoechst content. n = 5‐6. F) Schematic illustrating the groups and procedures of the mouse model. C57BL/6J mice were fed with vehicle or glutamine (2.1 g L‐Gln/kg body weight) under pair or ethanol feeding for 4 weeks. n = 6–9 biologically independent mice per group. G) Change curves of body weight of mice in the indicated group from (F). H) Liver TG levels in the indicated group from (F); n = 6–9. I) Representative H&E staining of liver sections in the indicated group in (F). Scale bars, 100 µm. J) RNA pol II activity of AML12 cells expressing pGL3‐Basic or pGL3‐Promoter treated with α‐Amanitin (10 µm) or glutamine (4 mm) in glutamine‐free medium for 12 h. Cells were serum starved overnight followed by ethanol (400 mm) stimulation for 1 h. n = 6–10. K) TG levels of the primary hepatocytes treated with α‐Amanitin (10 µm) or glutamine (4 mm) and ethanol (600 mm) in glutamine‐free medium for 24 h. The amount was normalized to the protein content. n = 4‐5. L) Bodipy levels of the primary hepatocytes described in (K). Scale bars, 50 µm. The amount was normalized to the hoechst content. n = 7–9. Data in (B–E), (J–L) are presented as the mean ± SEM, determined by one‐way ANOVA and Fisher's LSD test. Data in (H) are presented as the mean ± SEM, determined by two‐way ANOVA and Fisher's LSD test.

To determine which amino acids alleviate alcohol‐induced hepatic steatosis through the modulation of RNA pol II activity, we established a screening system involving a luciferase reporter assay and BODIPY assay (**Figure** **2**A). AML12 cells were cultured with different amino acids individually, revealing that 13 of these amino acids could reduce the RNA pol II activity induced by alcohol (Figure 2B). Then, the primary hepatocytes were isolated and treated with 13 amino acids individually, which revealed that only glutamine could decrease alcohol‐induced lipid accumulation (Figure 2C). Compared with healthy control mice, AFLD mice presented lower concentrations of glutamine (Figure S4A,B, Supporting Information), and the glutamine content in primary hepatocytes was decreased by ethanol treatment in a dose‐dependent manner (Figure S4C, Supporting Information).

To confirm that glutamine, but not its metabolite glutamate, can prevent alcohol‐induced lipid accumulation, we cultured primary hepatocytes in glutamine‐deprived medium and then treated them with glutamine or glutamate. Glutamate failed to alleviate alcohol‐induced lipid accumulation, indicating that only glutamine had this effect (Figure 2D,E). To assess the role of glutamine in alcohol‐induced fatty liver in vivo, a mouse model was created through the administration of glutamine along with alcohol for 4 weeks (Figure 2F). The food intake was kept constant, and the body weight curves remained similar (Figure 2G; Figure S4D, Supporting Information). A reduction in the serum TG concentration was observed in the glutamine‐treated mice (Figure S4E, Supporting Information). Compared with the alcohol‐fed group, the glutamine‐fed group presented a reduction in fatty liver, as evidenced by a reduction in the hepatic TG content (Figure 2H) and H&E staining (Figure 2I) of liver tissues. Additionally, the expression of lipogenic genes and proteins was decreased by glutamine (Figure S4G,H, Supporting Information). Furthermore, we detected the transcriptional effects of RNA pol II by hepatic RNA sequencing and found that RNA pol II transcriptional pathway gene expression was decreased by glutamine treatment (Figure S4F, Supporting Information).

To investigate whether glutamine in a high‐protein diet plays a key role in attenuating alcohol‐induced hepatic steatosis, we established an AFLD mouse model by feeding a normal diet, high‐protein diet or glutamine‐deficient high‐protein diet (Figure S5A, Supporting Information) with the administration of 5% ethanol for 2.5 weeks. The food intake was kept constant, and the body weight curves remained similar (Figure S5B,C, Supporting Information). Compared with that in high‐protein diet‐fed mice, the reduction in serum TG was abolished in glutamine‐deficient high‐protein diet‐fed mice (Figure S5D, Supporting Information). In the liver, the protective effects of the high‐protein diet on hepatic TG levels were abolished in the glutamine‐deficient high‐protein diet‐fed mice (Figure S5E,F, Supporting Information). Similarly, the inhibitory effects of a high‐protein diet on lipogenic genes and proteins were also abolished in the glutamine‐deficient high‐protein diet‐fed mice (Figure S5G,H, Supporting Information). To investigate whether glutamine prevents alcohol‐induced hepatic steatosis through RNA pol II, we examined the effects of glutamine on the inhibition of RNA pol II activity. Glutamine deprivation in hepatocytes significantly increased RNA pol II activity and TG production, and both of these effects were completely blocked by the RNA pol II inhibitor α‐amanitin (Figure 2J–L; Figure S4I, Supporting Information). Thus, these results indicate that the effects of glutamine on alcohol‐induced hepatic steatosis are dependent on RNA pol II.

### 2.3 Glutamine Stabilizes GLS1 and Reduces Alcohol‐Induced Hepatic Steatosis

**Figure 3:**
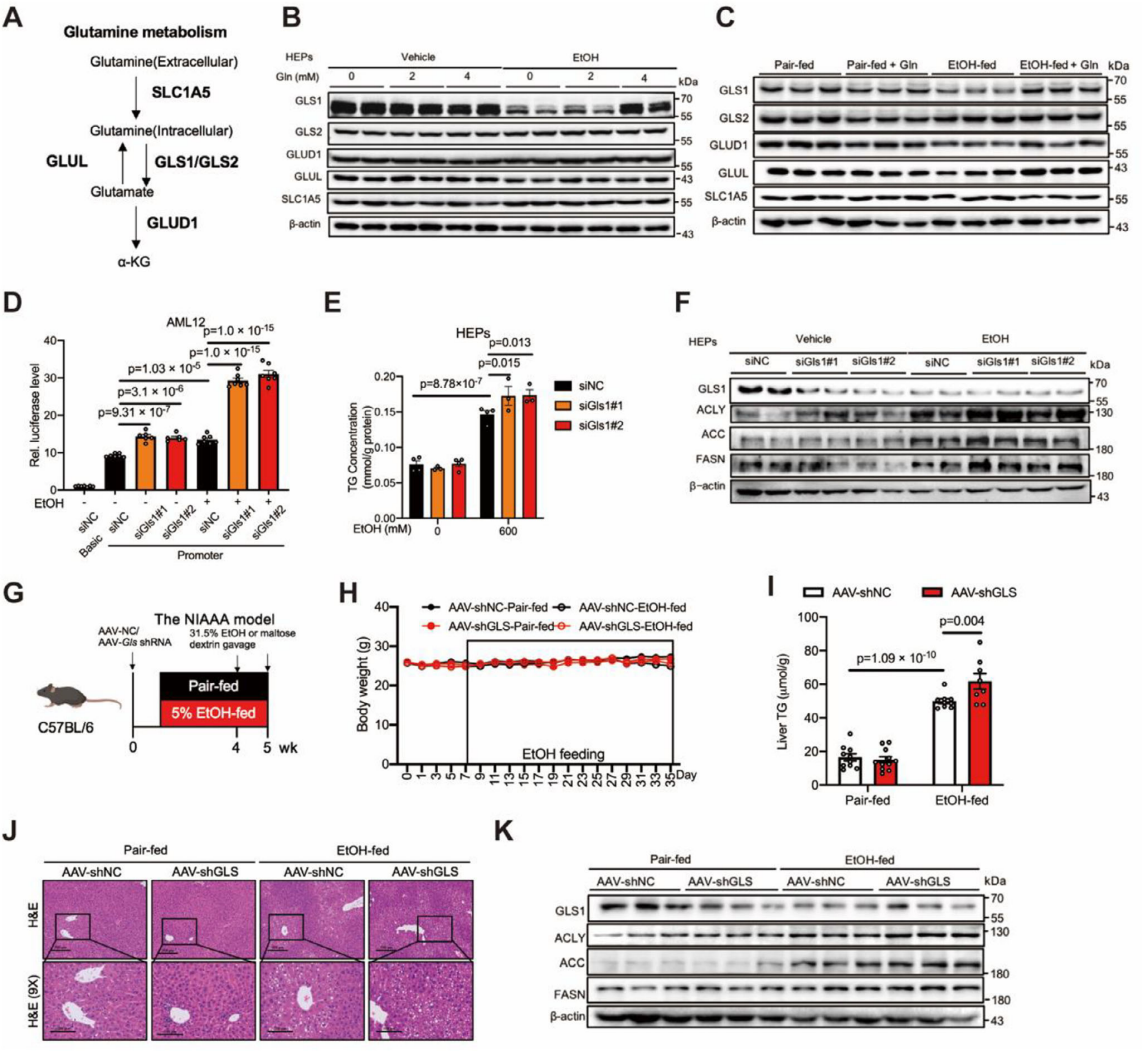
GLS1 stabilized by glutamine and liver‐specific knockdown of GLS1 increases hepatic alcoholic steatosis. A) A schematic diagram illustrating the glutamine metabolism. B) Western blots of representative genes of glutamine metabolism pathways in the primary hepatocytes treated with glutamine (2 or 4 mm) and ethanol (600 mm) for 24 h. β‐actin serves as a loading control. C) Western blots of representative genes of glutamine metabolism pathways of vehicle and glutamine‐fed mice under the pair or EtOH fed. β‐Actin serves as a loading control. D) RNA pol II activity of AML12 cells expressing pGL3‐Basic or pGL3‐Promoter with siRNAs targeting GLS1 or control. Cells were serum starved overnight followed by ethanol (400 mm) stimulation for 1 h. n = 6‐7. E) TG levels of the primary hepatocytes transfected with siRNAs targeting GLS1 or control. Cells were serum starved overnight followed by ethanol (600 mm) stimulation for 24 h. The amount was normalized to the protein content. n = 3‐4. F) Western blots of ACLY, ACC, FASN levels in the primary hepatocytes described in (E); β‐actin serves as a loading control. G) Schematic illustrating the groups and procedures of the mouse model. C57BL/6J mice were injected with AAV8‐TBG‐shGLS (AAV‐shGLS group) or AAV8‐TBG‐shNC (AAV‐shNC group) via the tail vein for additional 4‐week pair or ethanol feeding. n = 10‐11 biologically independent mice per group. H) Change curves of body weight of mice in the indicated group in (G). I) Liver TG levels of mice in the indicated group in (G); n = 8–11. J) Representative H&E staining of liver sections in the indicated group in (G). Scale bars, 100 and 200 µm. K) Western blots of ACLY, ACC, FASN levels in liver of the mice described in (G); β‐actin served as the loading control. Data in (D‐E), (I) are presented as the mean ± SEM, determined by two‐way ANOVA and Fisher's LSD test.

The effects of glutamine on alcohol‐induced hepatic steatosis, independent of its metabolite glutamate, led us to explore the underlying mechanism. We investigated proteins involved in glutamine metabolism‐associated pathways, including GLS1, GLS2, glutamate dehydrogenase 1 (GLUD1), glutamate‐ammonia ligase (GLUL), and solute carrier family 1 member 5 (SLC1A5) (**Figure** **3**A). Among these genes, only the expression of GLS1 was altered. The expression of GLS1 was dramatically decreased in alcohol‐treated hepatocytes and mice, whereas the inhibitory effect of alcohol on GLS1 was dependent on glutamine supplementation (Figure 3B,C; Figure S6, Supporting Information).

**Figure 4:**
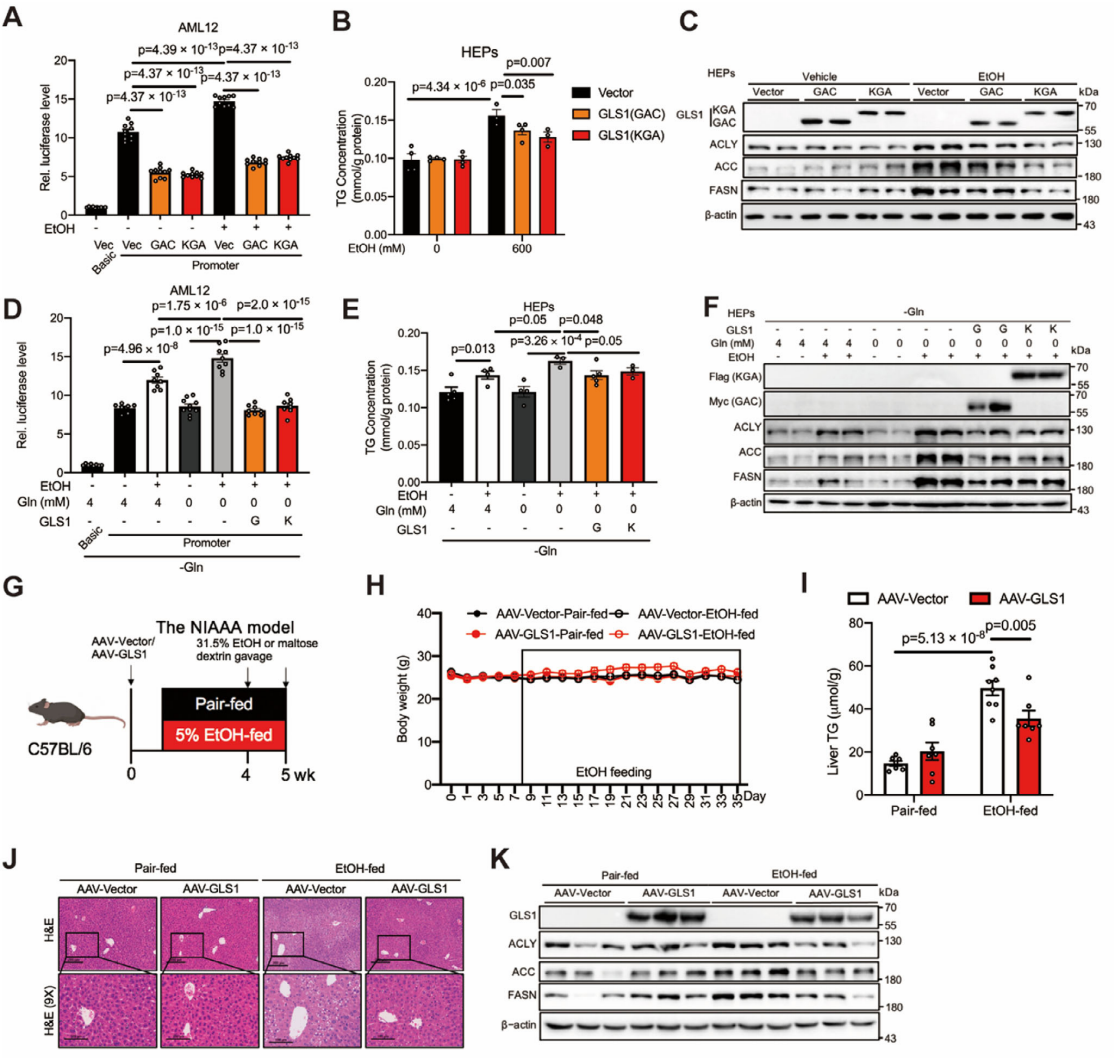
Liver‐specific over‐expression of GLS1 reduces hepatic alcoholic steatosis. A) RNA pol II activity of AML12 cells expressing pGL3‐Basic or pGL3‐Promoter with GLS1. Cells were serum starved overnight followed by ethanol (400 mm) stimulation for 1 h. n = 10. B) TG levels of the primary hepatocytes expressing GLS1. Cells were serum starved overnight followed by ethanol (600 mm) stimulation for 24 h. The amount was normalized to the protein content. n = 3‐4. C) Western blots of ACLY, ACC, FASN levels in the primary hepatocytes described in (B); β‐actin serves as a loading control. D) RNA pol II activity of AML12 cells expressing pGL3‐Basic or pGL3‐Promoter with GLS1. Cells were serum starved overnight in glutamine‐free medium followed by ethanol (400 mm) stimulation for 1 h. n = 7–10. E) TG levels of the primary hepatocytes expressing GLS1. Cells were serum starved overnight in glutamine‐free medium followed by ethanol (600 mm) stimulation for 24 h. The amount was normalized to the protein content. n = 3–5. F) Western blots of ACLY, ACC, FASN levels in the primary hepatocytes described in (E); β‐actin serves as a loading control. G) Schematic illustrating the groups and procedures of the mouse model. C57BL/6J mice were injected with AAV8‐TBG‐GLS (AAV‐GLS group) or AAV8‐TBG‐Vector (AAV‐Vector group) via the tail vein for additional 4‐week pair or ethanol feeding. n = 10–12 biologically independent mice per group. H) Change curves of body weight of mice in the indicated group in (G). I) Liver TG levels in the indicated group in (G); n = 7‐8. J) Representative H&E staining of liver sections in the indicated group in (G). Scale bars, 100 and 200 µm. K) Western blots of ACLY, ACC, FASN levels in liver of the mice described in (G); β‐actin served as the loading control. Data in (A‐B), (I) are presented as the mean ± SEM, determined by two‐way ANOVA and Fisher's LSD test. Data in (D‐E) are presented as the mean ± SEM, determined by one‐way ANOVA and Fisher's LSD test.

To assess the role of GLS1, we determined RNA pol II activity and lipid accumulation in hepatocytes. In the AML12 cell line, we found that GLS1 knockdown by siRNA exacerbated alcohol‐induced RNA pol II activity (Figure 3D; Figure S7A, Supporting Information). Additionally, the intracellular TG content was dramatically increased under conditions of GLS1 knockdown in hepatocytes (Figure 3E; Figure S7B, Supporting Information). Conversely, the overexpression of the GLS1 isoforms KGA and GAC resulted in a significant decrease in RNA pol II activity in AML12 cells and in the intracellular TG content in hepatocytes (**Figure** **4A,B**; Figure S8A,B, Supporting Information). Similarly, the expression of lipogenic genes and proteins in hepatocytes was induced in GLS1‐knockdown hepatocytes but decreased in GLS1‐overexpressing hepatocytes (Figure 3, 4; Figures S7C and S8E, Supporting Information). Next, we examined whether the effects of GLS1 on alcohol‐induced RNA pol II activity and TG content were affected by glutamine. In AML12 cells under glutamine deprivation conditions, overexpression of GLS1 also resulted in a significant decrease in RNA pol II activity (Figure 4D; Figure S8C, Supporting Information). The effects on the intracellular TG content and lipogenic proteins were similar in hepatocytes overexpressing GLS1 under glutamine deprivation conditions (Figure 4E,F; Figure S8D, Supporting Information). Similarly, the inactive GLS1 isoform also affected RNA pol II activity, TG content, lipogenic gene and protein expression (Figure S9, Supporting Information).

To further investigate the role of GLS1 in vivo, we created a mouse model with liver‐specific overexpression/knockdown of GLS1 and fed the mice a normal diet with or without alcohol (Figures 3G and 4G). The food intake was kept constant (Figures S7D and S8F, Supporting Information), and body weight curves remained similar across groups (Figures 3H and 4H).

Compared with control mice, hepatic GLS1 deficient mice presented aggravated fatty liver, as indicated by H&E staining (Figure 3J) of liver tissues and increased hepatic TG content (Figure 3I). Additionally, the expression of lipogenic genes and proteins was increased under GLS1 deficiency (Figure 3K; Figure S7F, Supporting Information). Furthermore, we detected the transcriptional effects of RNA pol II by hepatic RNA sequencing and found increased RNA pol II transcriptional pathway gene expression (Figure S7E, Supporting Information).

In contrast, the overexpression of GLS1 in the liver resulted in significantly decreased hepatic steatosis, as evidenced by decreased hepatic TG contents (Figure 4I), improved liver histology observed via H&E staining (Figure 4J), and decreased lipogenic gene and protein levels (Figure 4K; Figure S8G, Supporting Information). Consistently, transcriptional analysis revealed a decrease in RNA pol II transcriptional pathway gene expression in livers overexpressing GLS1 (Figure S8H, Supporting Information). Taken together, our data indicate that GLS1 is involved in the process of alcohol‐induced fatty liver.

To delineate the precise transcriptional step(s) at which GLS1 modulates RNA pol II activity, we performed a genome‐wide RNA pol II ChIP‐seq assay in AML12 cells and compared ethanol‐treated controls expressing an empty vector (EtOH‐vector) with their GLS1‐overexpressing counterparts (EtOH‐GLS1) (Figure S10A–C, Supporting Information). ChIP‐seq analysis revealed that GLS1 overexpression altered the RNA pol II distribution (Figure S10D, Supporting Information). In EtOH‐vector cells, 37.62% of the RNA pol II‐binding sites were mapped between genes (intergenic), whereas 9.54% were promoter regions, and 52.84% were intragenic; of these, 2.26% were within the 3′UTR, and 1.11% were within the 5′UTR (Figure S10E, Supporting Information). In EtOH‐GLS1 cells, 39.36% of the RNA pol II‐binding sites were mapped between genes (intergenic), whereas 8.48% were promoter regions, and 52.16% were intragenic; of these, 2.29% were within the 3′UTR, and 1.07% were within the 5′UTR (Figure S10F, Supporting Information). A comparison of these data revealed that at transcription initiation, the reduction in RNA pol II binding at promoter regions (−11.1%) indicated compromised transcription initiation efficiency. A decrease in the pausing index (−9.5%) was a secondary effect of initiation impairment. A reduction in intronic regions (−1.4%) indicated an overall reduction in transcriptional throughput, with CDS regions showing a concordant decrease. An increase in downstream regions (+2.6%) indicated a failure to properly disengage at transcription termination sites. Additionally, ChIP‐seq analysis of EtOH‐vector samples and EtOH‐GLS1 samples further validated the decreased binding of RNA pol II by GLS1 overexpression (Figure S10G, Supporting Information).

### 2.4 GLS1 Reduces RNA pol II Activity by Interacting with POLR2H or POLR2E

**Figure 5:**
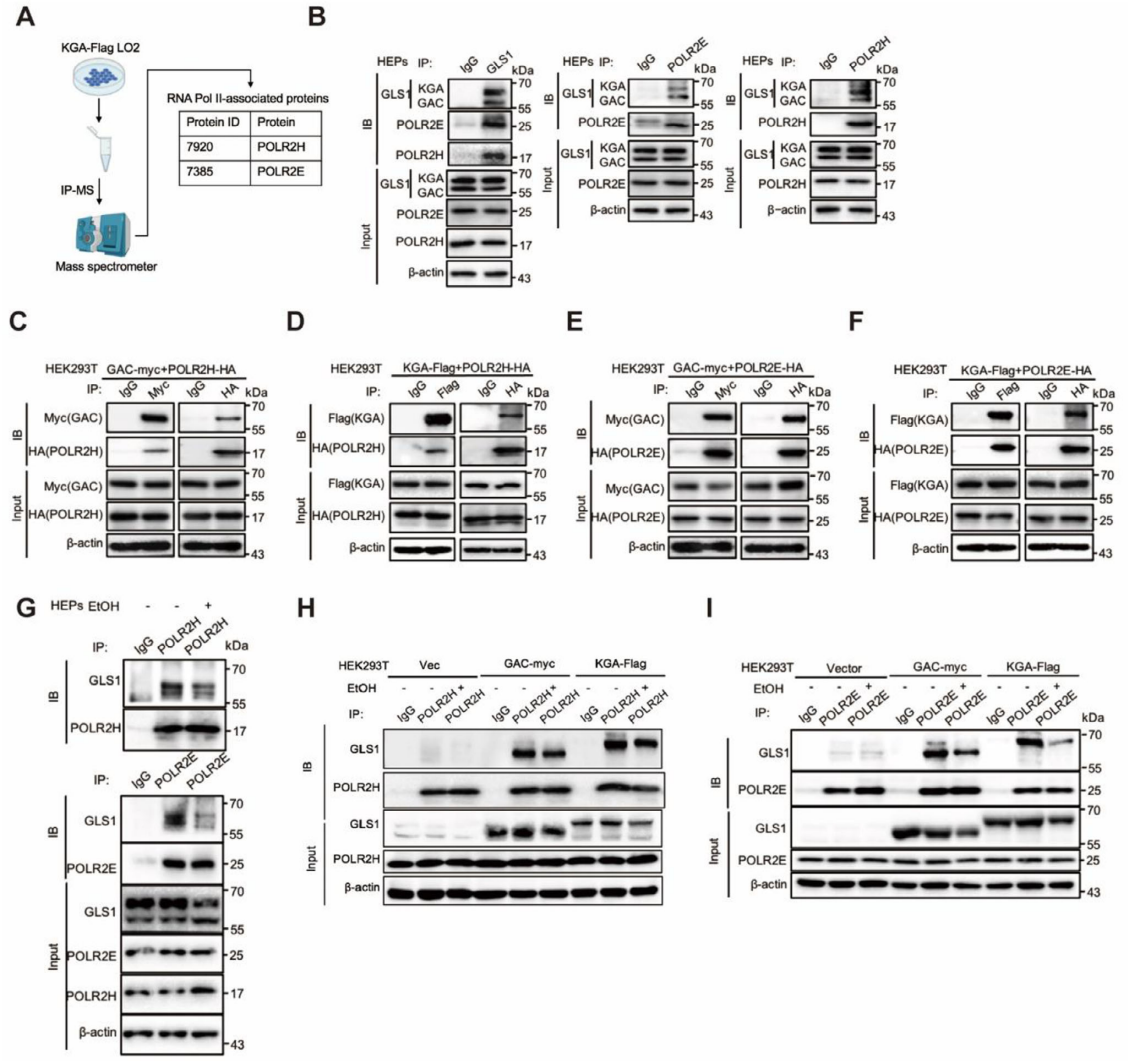
GLS1 interacts with POLR2E or POLR2H. A) Schematic of immunoprecipitation using mass spectrometry (IP–MS) to identify proteins associated with GLS1 in LO2 cells. B) CoIP analysis of the interaction of endogenous GLS1 with endogenous POLR2E or POLR2H in the primary hepatocytes. β‐actin served as the loading control. C–F) CoIP analysis of the interaction of GAC–Myc and POLR2H‐HA, KGA‐Flag and POLR2H‐HA, GAC–Myc and POLR2E‐HA and KGA‐Flag and POLR2E‐HA in HEK293T cells. β‐actin served as the loading control. G) CoIP analysis of the interaction of endogenous GLS1 with endogenous POLR2E or POLR2H in the primary hepatocytes treated with ethanol (600 mm) for 24 h. β‐actin served as the loading control. H,I) CoIP analysis of the interaction of GAC–Myc or KGA‐Flag with endogenous POLR2H, GAC–Myc or KGA‐Flag with endogenous POLR2E treated with ethanol (400 mm) for 24 h in HEK293T cells. β‐actin served as the loading control.

Next, we determined how GLS1 regulated RNA pol II. We performed Flag affinity immunoprecipitation (IP) of KGA‐Flag cells to identify binding partners for GLS1. Among the RNA polymerase components, POLR2E and POLR2H were identified in the KGA‐Flag IP elution, confirming that they were GLS1‐binding partners (**Figure** **5**A). Further validation using co‐IP analysis of hepatocyte/LO2/AML12 extracts revealed that endogenous GLS1 indeed interacted with endogenous POLR2E or POLR2H (Figure 5B; Figure S11A,B, Supporting Information). Additionally, in HEK293T cells, co‐overexpression of recombinant GAC‐myc or KGA‐Flag with POLR2H‐HA or POLR2E‐HA confirmed reciprocal interactions via FLAG or HA affinity IP analysis (Figure 5C–F). In hepatocytes, with or without GLS1 overexpression, alcohol did not affect the protein expression of POLR2E or POLR2H (Figure S11C, Supporting Information). Next, we investigated whether the interaction between GLS1 and POLR2E or POLR2H was affected by alcohol. Compared with the control, the interaction between endogenous GLS1 and endogenous POLR2E or POLR2H was decreased in hepatocytes treated with alcohol (Figure 5G). Similarly, we found that the interaction between recombinant KGA‐Flag or GAC‐myc and endogenous POLR2E or POLR2H was decreased in HEK293T cells treated with alcohol (Figure 5H,I).

**Figure 6:**
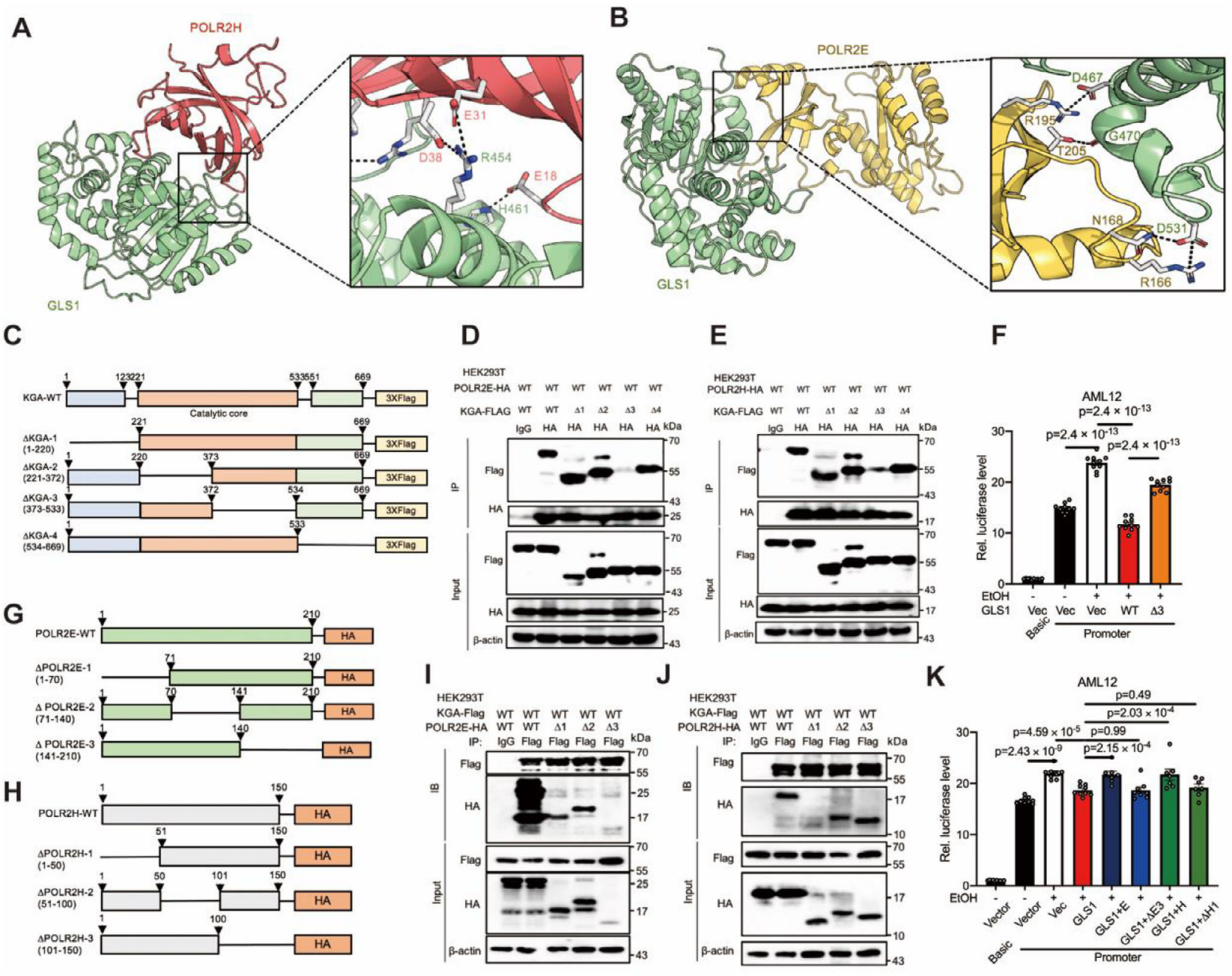
GLS1 interacts with POLR2H or POLR2E to reduce the activity of RNA pol II. A,B) The protein complex structure of GLS1, POLR2H and POLR2E performed on the GROMACS platform. The corresponding location of the putative binding motif on GLS1 and POLR2H protein, GLS1 and POLR2E protein. (C) The truncated GLS1 variants. D,E) CoIP analysis of the interaction of truncated GLS1 proteins and POLR2E‐HA or POLR2H‐HA in lysates of HEK293T cells. β‐actin served as the loading control. F) RNA pol II activity of AML12 cells expressing pGL3‐Basic or pGL3‐Promoter with KGA WT or ΔKGA‐3. Cells were serum starved overnight followed by ethanol (400 mm) stimulation for 1 h. n = 10. G,H) The truncated POLR2E or POLR2H variants. I,J) CoIP analysis of the interaction of truncated POLR2E or POLR2H proteins and KGA‐Flag in lysates of HEK293T cells. β‐actin served as the loading control. K) RNA pol II activity of AML12 cells expressing pGL3‐Basic or pGL3‐Promoter with GLS1 and POLR2E WT or ΔPOLR2E‐3 or POLR2H WT or ΔPOLR2H‐1. Cells were serum starved overnight followed by ethanol (400 mm) stimulation for 1 h. n = 7–10. Data in (F) and (K) are presented as the mean ± SEM, determined by one‐way ANOVA and Fisher's LSD test.

We subsequently examined the domains involved in the interaction between GLS1 and POLR2E or POLR2H. We conducted all‐atom MD simulations on the GROMACS platform. GLS1 was predicted to interact with POLR2H mainly by R454‐E31, R454‐D38 and H461‐E18 and with POLR2E mainly by D467‐R1952, G470‐T205, D531‐N168 and R166 (**Figure** **6**A,B). To validate these findings, we designed a series of truncated GLS1‐Flag variants (Figure 6C) and co‐overexpressed them with HA‐tagged POLR2E or POLR2H in HEK293T cells. HA‐based IP analysis revealed that all but the ∆KGA‐3 variant (amino acids 373–533 were truncated) were detected in the samples eluted from the cells, suggesting that GLS1 binds POLR2E or POLR2H through domain 3, which contains almost all the predicted GLS1‐binding sites (Figure 6D,E). Furthermore, RNA pol II activity decreased after the overexpression of KGA WT and, conversely, increased upon the overexpression of ∆KGA‐3 in AML12 cells (Figure 6F; Figure S12A, Supporting Information). Similarly, we designed truncated variants of POLR2E–HA and POLR2H–HA (Figure 6G,H) and co‐overexpressed them with Flag‐tagged GLS1 in HEK293T cells. Flag‐based IP analysis revealed that all but the ∆POLR2E‐3 variant (amino acids 141–210 were truncated) or the ∆POLR2H‐1 variant (amino acids 1–50 were truncated) were detected in the samples eluted from the cells, suggesting that POLR2E binds GLS1 through domain 3 (Figure 6I) and that POLR2H binds GLS1 through domain 1 (Figure 6J), which encompasses the predicted binding sites. Furthermore, GLS1 reduced RNA pol II activity, which was impeded in cells expressing POLR2E or POLR2H WT compared with their variant‐expressing counterparts (Figure 6K; Figure S12D, Supporting Information). Thus, these data demonstrate that GLS1 interacts with POLR2H or POLR2E and that this interaction is involved in the regulation of the RNA pol II complex.

To investigate the subcellular locations of GLS1, POLR2E and POLR2H, we subjected primary hepatocytes to nuclear and cytoplasmic extraction and found that GLS1, POLR2E and POLR2H were present both in the cytoplasm and nucleus (Figure S11D, Supporting Information). In addition, to investigate the location of the interaction between GLS1 and POLR2E or POLR2H, co‐IP analysis of the interaction of KGA‐Flag with endogenous POLR2H or POLR2E after nuclear and cytoplasmic extraction from HEK293T cells was performed. We found that in the nucleus, there was a physical interaction between GLS1 and POLR2E or POLR2H (Figure S11E, Supporting Information). While GLS1 possesses structural features necessary for mitochondrial targeting,^[^ ^30^ ^]^ it lacks a conventional nuclear localization signal. However, GLS1 has other sequence motifs and conserved modules that might be essential for its nuclear import.^[^ ^31^ ^]^ For example, exon 1 of human GLS1 contains an LXXLL signature motif,^[^ ^32^ ^]^ which might explain the nuclear localization recently demonstrated for glutaminase.^[^ ^33^ ^]^ Therefore, we constructed a KGA^∆L^ plasmid in which the LXXLL signature motif was deleted (Figure S11F, Supporting Information). We found that KGA^∆L^ was less present in the nucleus than was KGA WT (Figure S11G, Supporting Information), and there was less interaction between KGA^∆L^ and POLR2E or POLR2H (Figure S11H, Supporting Information). Similarly, KGA^∆L^ did not affect RNA pol II activity, TG content, lipogenic gene and protein levels (Figure S11I–L, Supporting Information).

### 2.5 The Interaction Between GLS1 and RNA Polymerase II is Accounted for Alcohol‐Induced Fatty Liver

**Figure 7:**
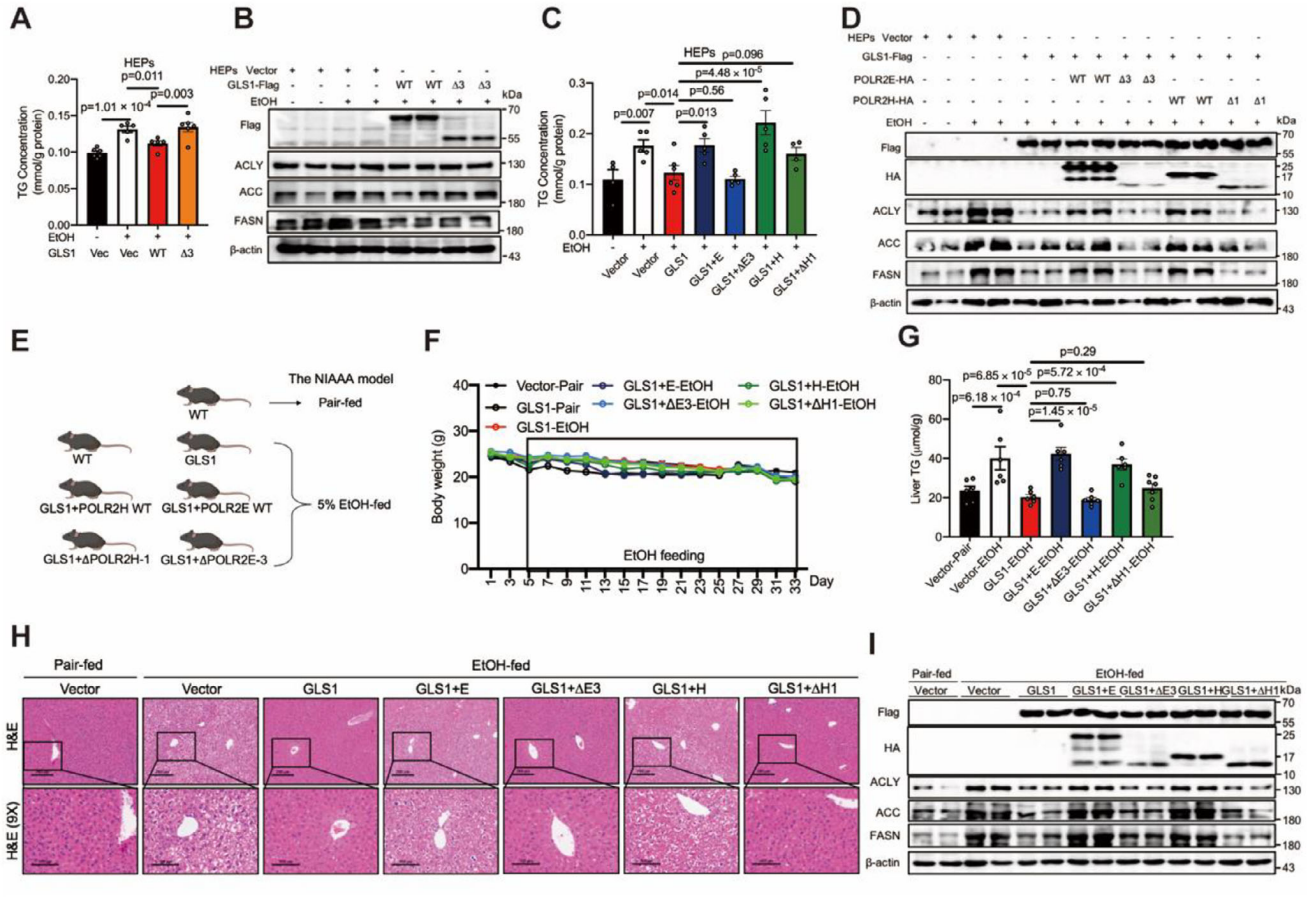
GLS1 reduces hepatic alcoholic steatosis through interacting with POLR2E or POLR2H. A) TG levels of the primary hepatocytes expressing with KGA WT or ΔKGA‐3. Cells were serum starved overnight followed by ethanol (600 mm) stimulation for 24 h. The amount was normalized to the protein content. n = 5‐6. B) Western blots of ACLY, ACC, FASN levels in the primary hepatocytes described in (A); β‐actin serves as a loading control. C) TG levels of the primary hepatocytes expressing with GLS1 and POLR2E WT or ΔPOLR2E‐3 or POLR2H WT or ΔPOLR2H‐1. Cells were serum starved overnight followed by ethanol (600 mm) stimulation for 24 h. The amount was normalized to the protein content. n = 4–6. D) Western blots of ACLY, ACC, FASN levels in the primary hepatocytes described in (C); β‐actin serves as a loading control. E) Schematic illustrating the groups and procedures of the mouse model. C57BL/6J mice were injected with AAV8‐TBG‐GLS1 with POLR2E WT or POLR2H WT or their truncated variants (ΔPOLR2E‐3 or ΔPOLR2H‐1) via the tail vein for additional 4‐week pair or ethanol feeding. n = 8–10 biologically independent mice per group. F) Change curves of body weight of mice. G) Liver TG levels of mice in the indicated group in (E); n = 6‐7. H) Representative H&E staining of liver sections in the indicated group in (E). Scale bars, 100 and 200 µm. I) Western blots of ACLY, ACC, FASN levels in the primary hepatocytes described in (E); β‐actin serves as a loading control. Data in (A), (C), (G) are presented as the mean ± SEM, determined by one‐way ANOVA and Fisher's LSD test.

To clarify the pathological function of GLS1‐binding domains in alcohol‐induced fatty liver, hepatocytes were transfected with GLS1 (KGA) WT or ∆KGA‐3. We observed that intracellular lipid accumulation was decreased in cells overexpressing GLS1 (KGA) WT but not in those overexpressing the ∆KGA‐3 variant (**Figure** **7**A; Figure S12B, Supporting Information). Consistently, the expression of lipogenic genes and proteins was inhibited in cells overexpressing GLS1 (KGA) WT but not the expression of the ∆KGA‐3 variant (Figure 7B; Figure S12C, Supporting Information). We also performed experiments to assess the effects of POLR2E and POLR2H. We overexpressed GLS1 with POLR2E WT or POLR2H WT or their truncated variants to assess whether GLS1 could reduce alcohol‐induced fatty liver. We observed that the reduction in intracellular lipid accumulation caused by GLS1 was impeded by POLR2E WT or POLR2H WT but not by their variants (Figure 7C; Figure S12E, Supporting Information). The decreased expression of lipogenic genes and proteins by GLS1 was induced by POLR2E WT or POLR2H WT but not by their variants (Figure 7D; Figure S12F, Supporting Information).

Based on the above results, we used a mouse model to detect the effects of the interaction sites on alcohol‐induced fatty liver. We established models co‐overexpressing GLS1 and POLR2E or POLR2H WT, alongside their truncated variants, in the liver (Figure 7E). Constant food intake was maintained, and the body weight curves were consistent across the experimental groups (Figure 7F; Figure S12G, Supporting Information). Compared with that in GLS1‐overexpressing mice, the reduction in the serum TG was abolished in mice overexpressing POLR2E WT or POLR2H WT (Figure S12H, Supporting Information). In the liver, the protective effect on hepatic TG content was impeded by POLR2E or POLR2H (Figure 7G,H). Similarly, the inhibitory effects of GLS1 on lipogenic gene and protein expression were also inhibited by POLR2E or POLR2H (Figure 7I; Figure S12I, Supporting Information). In contrast, the overexpression of GLS1 with truncated variants of POLR2E or POLR2H did not affect the protective effects of GLS1 on alcohol‐induced fatty liver (Figure 7G–I). Collectively, these findings demonstrate that in the liver, GLS1 ameliorates alcohol‐induced fatty liver through its interaction with POLR2H or POLR2E.

## 3 Conclusion

**Figure 8:**
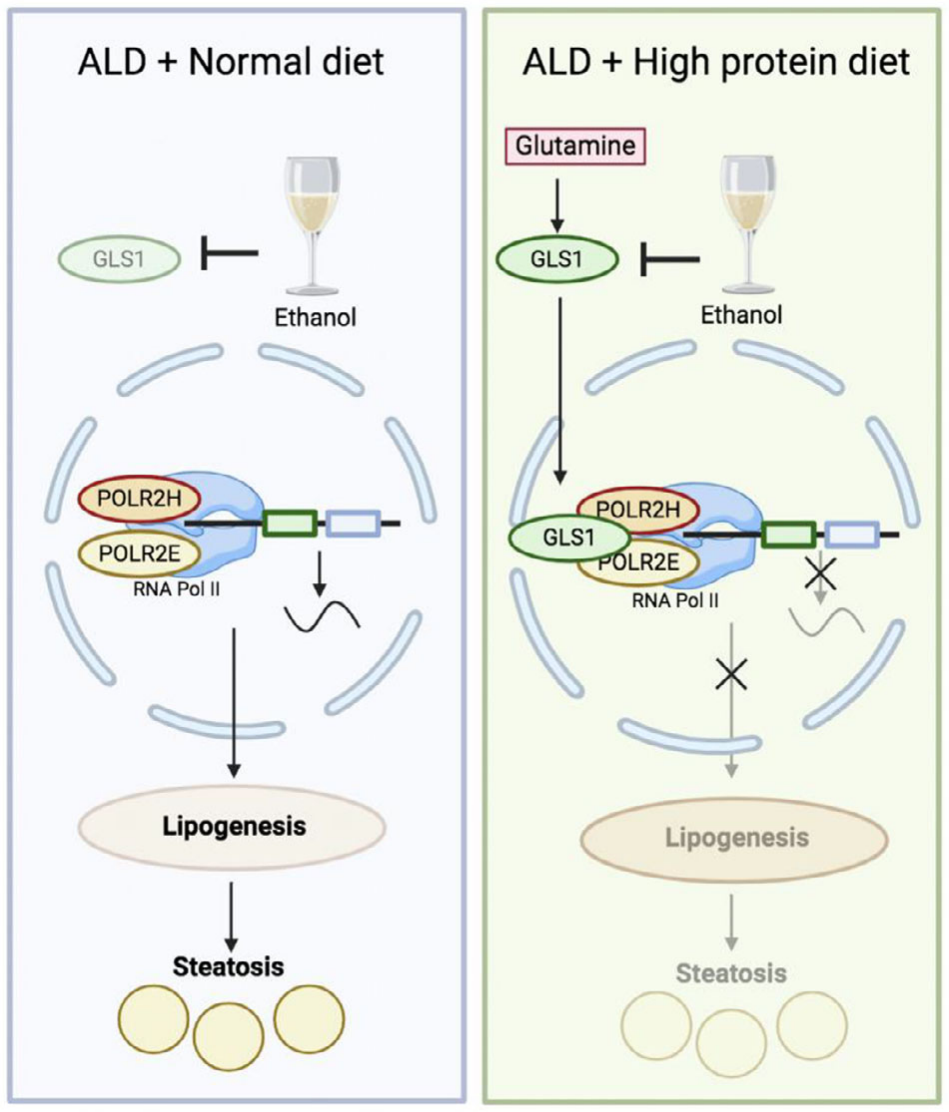
Schematic diagram of glutamine‐regulated GLS1 coordinates RNA polymerase II manipulates AFLD. High‐protein diet reduces alcoholic hepatic steatosis through glutamine stabilizing GLS1 to regulate RNA pol II activity by interacting with POLR2E or POLR2H.

Our study reveals a previously unrecognized regulatory mechanism in which glutaminase 1 (GLS1) modulates RNA polymerase II activity, providing a molecular foundation for understanding the hepatoprotective effects of a high‐protein diet in alcoholic liver disease (AFLD) (**Figure** **8**).

Current research presents complex metabolic alterations in AFLD. Previous work by Choi et al. reported alcohol‐induced dysregulation of glutamate biosynthesis‐related genes, including the induction of hepatic aldehyde dehydrogenase 4 family member a1 (Aldh4a1) and the reduction of glutamate‐ammonia ligase (Glul), leading to glutamate accumulation and glutamine reduction.^[^ ^34^ ^]^ However, in our study, we observed alcohol‐mediated destabilization of the GLS1 protein despite unchanged mRNA levels. This posttranslational regulation is evidenced by the promotion of GLS1 degradation in cycloheximide‐treated hepatocytes (Figure S6C, Supporting Information). The precise mechanisms of degradation, including potential ubiquitination pathways and the mechanism by which glutamine restores GLS1 levels, require further investigation. Previous studies have demonstrated that glutamine exerts antioxidant effects, including increasing glutathione (GSH) levels and scavenging reactive oxygen species (ROS), which are pathogenic factors in the progression of AFLD.^[^ ^34^ ^]^ In our study, we demonstrate that glutamine can stabilize GLS1, leading to the inhibition of RNA pol II activity, which contributes to AFLD development. The regulation of ROS primarily occurs in the cytoplasm, while the effects of glutamine on RNA pol II are nuclear, suggesting that both cytoplasmic and nuclear effects may contribute to the overall hepatoprotection afforded by glutamine.

While GLS1 is located predominantly in mitochondria, its nuclear localization and catalytic activity are well documented.^[^ ^33^, ^35^ ^]^ Notably, we confirmed the functional significance of nuclear GLS1 compartmentalization. Nuclear glutaminase has distinct kinetic properties,^[^ ^33^ ^]^ and emerging evidence supports noncanonical transcriptional regulatory functions.^[^ ^31^, ^36^ ^]^ Our study demonstrates a direct interaction between nuclear GLS1 and the POLR2H/POLR2E subunits of RNA pol II. Although GLS1 contains mitochondrial targeting sequences,^[^ ^30^ ^]^ it lacks a canonical nuclear localization signal (NLS). However, an evolutionarily conserved LXXLL signature in exon 1 of human GLS1 may facilitate nuclear import.^[^ ^32^ ^]^ This motif has been mechanistically linked to nuclear glutaminase localization.^[^ ^33^ ^]^ To test this hypothesis, we generated a KGA ΔL mutant with targeted deletion of the LXXLL motif. Subcellular fractionation significantly reduced the nuclear accumulation of KGA ΔL compared with that of KGA WT. Moreover, coimmunoprecipitation revealed a reduction in the interaction of KGA ΔL with both POLR2E and POLR2H. This localization defect translated to functional impairment. KGA ΔL failed to suppress RNA pol II activity and had no significant effect on lipogenic gene or protein expression or triglyceride accumulation induced by ethanol. Thus, nuclear GLS1 is important for alcohol‐induced fatty liver.

RNA pol II has been implicated in the regulation of lipid metabolism.^[^ ^24^, ^25^ ^]^ For example, RNA pol II plays an important role in the transcription and splicing of lipogenesis‐related enzymes^[^ ^26^ ^]^ and mitochondrial biogenesis‐related genes,^[^ ^27^ ^]^ leading to hepatic lipogenesis. During AFLD, increased RNA pol II activity contributes to hepatic steatosis caused by alcohol. A high‐protein diet, which is commonly used clinically to alleviate AFLD, can reduce RNA pol II activity. Furthermore, we investigated the mechanistic basis for ethanol‐induced RNA pol II activation. Our findings demonstrate that GLS1 interacts with the RNA pol II subunits POLR2H and POLR2E to modulate RNA pol II transcriptional activity, thereby promoting hepatic steatosis in AFLD. This discovery reveals a previously unrecognized regulatory mechanism through which metabolic enzymes can directly influence RNA pol II‐dependent gene expression programs in liver disease.

While we utilized α‐amanitin (AMA) as a pharmacological tool to detect the involvement of RNA pol II in the pathogenesis of AFLD, we emphasize its inherent limitations as a clinically acceptable therapeutic candidate. In our study, AMA was administered at a low dose (0.15 mg kg^−1^, i.p., every other day), which was carefully maintained below established toxicity thresholds. Notably, chronic AMA treatment did not induce observable adverse effects in our mouse model, confirming that the observed amelioration of alcohol‐induced fatty liver by AMA is not confounded by systemic toxicity. As anticipated, AMA treatment significantly attenuated alcohol‐induced hepatic steatosis, supporting the premise that RNA pol II inhibition can mitigate AFLD progression. However, AMA lacks liver specificity as an RNA pol II inhibitor, and its irreversible suppression of this essential transcriptional machinery in all cell types results in prohibitive systemic toxicity, rendering it clinically unsuitable. Given the challenges of genetically manipulating RNA pol II, a universally required transcriptional enzyme, our use of AMA is strictly limited to establishing causality between RNA pol II activity and alcohol‐induced steatosis and to demonstrating that the hepatoprotective effects of glutamine require functional RNA pol II. Collectively, these findings position the GLS1‐RNA pol II axis as a novel mechanistic pathway worthy of further investigation, particularly for the development of tissue‐specific modulators for AFLD therapy.

In summary, we delineate a novel pathway in which dietary glutamine stabilizes GLS1, enabling its nuclear interaction with POLR2H/POLR2E to suppress RNA pol II activity and attenuate alcohol‐induced hepatic steatosis. Our research establishes a crucial mechanistic link between amino acid metabolism and transcriptional regulation, providing foundational insights for future therapeutic approaches in AFLD.

## 4 Experimental Section

### Animals, Diet and Drug Treatment

All animal experiments were authorized by the Institutional Animal Care and Use Committee at the Shanghai Institute of Materia Medica. The assigned approval numbers of the ethical approval for animal experiments were 2022‐08‐LJ‐134, 2023‐08‐LJ‐157 and 2025‐04‐LJ‐205. C57BL/6J mice (male, 19–22 g, 5‐week‐old) were purchased from Shanghai SLAC Laboratory Animal Company and housed under specific‐pathogen‐free conditions in Shanghai Institute of Materia Medica. The mice were maintained on a 12 h light–dark cycle at 22 ± 0.5 °C, 50–60% humidity, with food and water provided ad libitum.

A simple murine model of AFLD (the NIAAA model) was performed by chronic ethanol feeding (3–5 weeks ad libitum oral feeding with the Lieber‐DeCarli ethanol or control liquid diet; Research Diets, Inc, USA) plus a binge ethanol feeding, which synergistically induced liver injury, inflammation and fatty liver as described.^[^ ^28^ ^]^ Composition of diets used in this study was provided in Table S1 (Supporting Information). Briefly, a NP pair diet containing 17% protein, 47% carbohydrate and 36% fat. A NP ethanol diet containing 17% protein, 11% carbohydrate, 36% fat and 36% ethanol. A HP ethanol diet containing 34% protein, 7% carbohydrate, 23% fat and 36% ethanol. To establish the glutamine‐feeding model, mice were fed with either an ethanol or control liquid diet with vehicle or glutamine (2.1 g L‐Gln kg^−1^ body weight, G3126; Sigma–Aldrich, USA) for 4–5 weeks. For pharmacological inhibition of RNA pol II, mice were treated with α‐Amanitin (0.15 mg kg^−1^ body weight, i.p., every other day, HY‐19610; MedChemExpress, China) for the last 2 weeks of ethanol feeding.

For liver‐specific over‐expression of GLS1, mice were injected with AAV8‐TBG‐GLS1, or AAV8‐TBG‐Vector as a control, into the tail vein at a dose of 1 × 10^11^ vg per mouse. For liver‐specific knockdown of GLS1, mice were injected with AAV8‐TBG‐shGLS1, or AAV8‐TBG‐shNC as a control, into the tail vein at a dose of 1 × 10^11^ vg per mouse. For AAV‐mediated liver co‐overexpression, mice were injected with AAV8‐TBG‐GLS1 with AAV8‐TBG‐POLR2E WT or AAV8‐TBG‐POLR2H WT or AAV8‐TBG‐ΔPOLR2E‐3 or AAV8‐TBG‐ΔPOLR2H‐1 via the tail vein at a dose of 1 × 10^11^ plus 1 × 10^11^ vg per mouse. The adenovirus was purchased from Jiman Biotechnology (China). Mice were fed with either an ethanol or control liquid diet for 4–5 weeks after AAV injection.

Plasma levels of total TG were determined by an Olympus AU 600 auto‐analyzer (Olympus, Japan), according to the manufacturer's instructions. H&E staining of liver samples were performed by Servicebio (China). Sections were imaged by Vectra automated quantitative pathology system (PerkinElmer), according to the manufacturer's instructions.

### Chemical Reagents, Antibodies, and Cell Lines

Chemical reagents, antibodies, and cell lines used were described in Table S3 (Supporting Information). The primary hepatocytes were isolated from the liver of C57/BL6 male mice, as previously reported.^[^ ^37^ ^]^ The isolated hepatocytes were then incubated overnight at 37 °C (95% relative humidity, 5% CO_2_) before further experiments. AML12 cells obtained from ATCC were cultured in Dulbecco's Modified Eagle Medium: F12 (DMEM/F12, 11320033; Gibco) supplemented with 10% fetal bovine serum (FBS; Gibco) and 1% penicillin/streptomycin (Gibco). HEK293T cells obtained from the Cell Bank of the Type Culture Collection Center of the Chinese Academy of Sciences were maintained in DMEM (11965167; Gibco) supplemented with 10% (v/v) FBS and 1% penicillin/streptomycin. LO2 cells obtained from ATCC were cultured in RPMI 1640 (11875101; Gibco) supplemented with 10% fetal bovine serum (FBS; Gibco) and 1% penicillin/streptomycin (Gibco). All cells were cultured in a humidified incubator at 37 °C with 5% CO_2_ before further experiments.

### Cell Treatment

To assess the response to ethanol, cells were treated with ethanol (100092008; Sinopharm Chemical Reagent Co.Ltd, China). For amino acids screening, cells were treated with 20 amino acids (MedChemExpress) for 12 or 24 h. To assess the response to glutamine and glutamate, cells were treated with glutamine or glutamate in DMEM deprived of glutamine (11054020; Gibco) for 12 or 24 h. The dose of amino acids added in cells refers to the concentration of the culture medium. For pharmacological inhibition of RNA pol II, cells were treated with α‐Amanitin (2, 10 µm) for 12 or 24 h.

### Plasmids Construct and Cell Transfection

For siRNA transfection, cells were transfected with control siRNA or GLS1 siRNA (Shanghai GenePharma Co.,Ltd, China) for 24 h using lipofectamine 2000 transfection reagent (Thermo Fisher Scientific, USA), according to the manufacturer's instructions.

For GLS1 overexpression, cells were transfected with control plasmids or GAC sequence with the Myc tag or KGA sequence with the 3 × Flag tag plasmids for 24 h using lipofectamine 3000 transfection reagent (Thermo Fisher Scientific). For co‐expression, cells were co‐transfected with control or KGA and POLR2E WT or ΔPOLR2E‐3 or their variants plasmids for 24 h using lipofectamine 3000 transfection reagent (Thermo Fisher Scientific), according to the manufacturer's instructions. GAC, KGA, POLR2H and POLR2E WT plasmids were obtained from Youbio company (China). The series of truncated KGA‐Flag or POLR2E–HA or POLR2H–HA variants and GLS1 mutation sequence was amplified using Primer STAR HS DNA Polymerase (Takara, Japan) from cDNA and inserted into pcDNA3.1 vector. Primer sequences for plasmids construction used were described in Table S2 (Supporting Information).

### Immunoprecipitation and Mass Spectrometry Analysis

Cells with overexpression FLAG‐Flag lysed by cell lysis IP buffer (P0013; Beyotime Biotechnology) were incubated overnight at 4 °C with Flag‐tag (8146; Cell Signaling Technology) antibody and protein A+G Agarose beads (P2055; Beyotime Biotechnology). Beads containing immune complexes were washed and precipitated protein samples were submitted to further MS analysis supported by Majorbio (China). The mass spectrometry proteomics data have been deposited to the ProteomeXchange Consortium via the PRIDE partner repository with the dataset identifier PXD050603.

### Co‐Immunoprecipitation

For analysis of endogenous proteins, the primary hepatocytes lysed by cell lysis IP buffer were incubated overnight at 4 °C with GLS1 (12855‐1; Proteintech) or POLR2E (sc‐390902; santa cruz) or POLR2H (sc‐398512; santa cruz) antibody and protein A+G Agarose beads. For analysis of recombinant proteins, HEK293T cells co‐expression with POLR2E–HA or POLR2H–HA together with GLS1 lysed by cell lysis IP buffer were incubated overnight at 4 °C with Flag‐tag or Myc‐tag (2276; Cell Signaling Technology) antibody or HA‐tag (3724; Cell Signaling Technology) antibody and protein A+G Agarose beads. Beads containing immune complexes were washed and collected. The input and precipitated protein samples were subjected to western blot analysis with corresponding antibodies as stated. Western blots of the elution of the control group were used to control for any non‐specific binding of the beads.

### Western Blotting Analysis

Cultured cells and mouse liver tissues were lysed by RIPA Lysis Buffer (P0013B; Beyotime Biotechnology) on ice for 30 min, followed by centrifugation. Supernatants were separated via SDS‐polyacrylamide gel electrophoresis. Proteins on the gel were subsequently transferred to a polyvinyl difluoride membrane (Bio‐Rad, USA). After blocking with the Tris‐buffered saline with 0.1% Tween 20 buffer (20 mm Tris, pH 8.0, 150 mm NaCl, and 0.1% Tween 20) containing 5% non‐fat milk for 2 h, the membranes were immunoblotted with primary antibodies at 4 °C overnight, followed by incubation with horseradish peroxidase‐conjugated secondary antibodies (Proteintech). Finally, an enhanced chemiluminescence reagent was used for signal detection.

### Bodipy Imaging

Specific imaging of lipid droplets in the primary hepatocytes were performed by using BODIPY‐based fluorogenic Probe. The primary hepatocytes were fixed by 4% paraformaldehyde. Working solution of the bodipy dyes (D3861, Thermo Fisher Scientific) were diluted to 5 µm with PBS, and used to stain the cells for 10 min. Working solution of the Hoechst 33342 dyes (S36939, Thermo Fisher Scientific) were diluted to 10 µg mL^−1^ with PBS, and used to stain the cells for 10 min. The bodipy levels of cells were imaged using a laser scanning confocal microscope (Operetta, PerkinElmer, UK), and normalized to the Hoechst 33342 content.

### Determination of Triacylglycerol Content

Measurements of triglyceride in mice liver were performed by using freshly isolated liver samples. Tissue was homogenized with 0.5 mL of phosphate‐buffered saline, and the total triglyceride was extracted with 1.5 mL of chloroform and methanol (2:1 v/v) overnight. The samples were centrifuged, and the bottom liquid phase was transferred to a clean tube and air‐dried. The triglyceride was then dissolved in 1 mL of ethanol containing 1% Triton X‐100. The triglyceride levels were determined using a triglyceride assay kit (Jiancheng bioengineering institute, China), and normalized to the tissue weight, according to the manufacturer's protocol. Measurements of triglyceride in the primary hepatocytes were performed by using freshly isolated cells. Cells were lysed by RIPA Lysis Buffer on ice for 30 min, followed by ultrasonic disruption and centrifugation. The triglyceride levels of supernatants were determined using a triglyceride assay kit (Jiancheng bioengineering institute), and normalized to the protein content, according to the manufacturer's protocol.

### Luciferase Reporter Assay

Equimolar amounts of pGL3‐basic plasmid or pGL3‐promoter plasmid (containing SV40 promoter) were transfected into AML12 cells using Lipofectamine 3000 (Thermo Fisher Scientific). After 48 h post‐transfection, luciferase activity was measured using Steady‐Lumi II Firefly Luciferase Assay Kit (RG059; Beyotime Biotechnology), according to the manufacturer's protocol. The cell viability was measured using CellTiter‐Glo Luminescent Cell Viability Assay (CTG, G7572; Promega), according to the manufacturer's protocol. The ratio of luciferase to CTG was calculated to represent the relative Luc activity.

### EU‐Click Assays

Specific imaging of overall transcription levels in the AML12 cells were performed by using BeyoClick EU RNA Synthesis Kit with Alexa Fluor 488(R0301S, Beyotime Biotechnology), according to the manufacturer's protocol. Working solution of the EU were diluted to 1 mm, and treated cells for 2 h. Cells were fixed by 4% paraformaldehyde for 15 min. Working solution of Click Additive Solution including Click Reaction Buffer, CuSO4, Azide 488 and Click Additive was prepared according to the manufacturer's protocol, and used to stain cells for 30 min. Working solution of the Hoechst 33342 dyes were diluted to 10 µg mL^−1^ with PBS, and used to stain cells for 10 min. The Azide 488 levels of cells were imaged using a laser scanning confocal microscope (Operetta, PerkinElmer, UK), and normalized to the Hoechst 33342 content.

### Nuclear and Cytoplasmic Extraction

Nuclear and cytoplasmic extraction of cells were performed by using Nuclear and Cytoplasmic Protein Extraction Kit (P0027; Beyotime Biotechnology), according to the manufacturer's protocol. In brief, cells were harvested with trypsin‐EDTA and then centrifuged. Cytoplasmic extract obtained by using CER I and CER II buffer, nuclear extract obtained by using NER buffer. The nucleus and cytoplasmic fractions protein samples were subjected to western blot analysis with corresponding antibodies as stated. Histone 3 and GAPDH served as a loading control for nucleus and cytoplasmic fractions, respectively.

### Quantitative Real‐Time PCR

Total RNA from mouse livers was isolated using the TRIzol method (Invitrogen, Shanghai, China). Total RNA from the primary hepatocytes were performed using freshly isolated cells and lysed using the TRIzol method (Invitrogen, Shanghai, China). The results were analyzed on an ABI StepOne Plus real‐time PCR system (Applied Biosystems, USA) using the 2‐∆∆Ct method. β‐actin was used as control, and relative mRNA levels were normalized to β‐actin. Primer sequences for qPCR used were described in Table S2 (Supporting Information).

### Chromatin Immunoprecipitation and Next Generation Sequencing (ChIP‐Seq)

For each condition, cells after treated were harvested from three independent samples. ChIP assays were performed using SimpleChIP Enzymatic Chromatin IP Kit (9003, Cell Signaling Technology) as described by manufacturers. In brief, after cross‐linking, nuclei were purified, and chromatin was sheared by sonication. Chromatin was incubated overnight with RNA pol II antibody (39097, Active Motif). Normal Rabbit IgG (2729, Cell Signaling Technology) were used as a negative control for the immunoprecipitation. Immunoprecipitated chromatin was then incubated with ChIP‐Grade Protein G Magnetic Beads (9006, Cell Signaling Technology), washed and eluted. After reversal of the cross‐links and purification of DNA, precipitated DNAs were analyzed by next generation sequencing. ChIP‐Seq libraries were constructed by end‐repairing, A‐tailing, and adapter ligating DNA fragments, followed by size selection and PCR amplification. Libraries underwent quality control using the Agilent 5400 system and were quantified to 1.5 nm by QPCR. Pooled libraries were sequenced on the NovaSeq X Plus platform (CHI BIOTECH CO., LTD) generating 150‐bp paired‐end reads using sequencing‐by‐synthesis chemistry. Raw data was processed with fastp (v0.23.4) to remove adapters, poly‐N, and low‐quality reads, generating clean data with calculated Q20/Q30/GC content. Clean reads were aligned to a reference genome using Bowtie2 (v2.5.1). Peak calling was performed with MACS2 (v2.2.6) using a q‐value threshold of 0.05. Identified peaks were annotated for gene functional regions and subjected to GO/KEGG enrichment analysis using CHIP seeker (v1.34.1) and cluster Profiler R (v4.6.2). Motif discovery on 500‐bp sequences centered at peak summits was conducted with HOMER (v4.11.1). Differential peak analysis between groups was performed using DiffBind (v3.8.4), followed by functional annotation and enrichment analysis of differential peaks with CHIP seeker and cluster Profiler. The ChIP‐seq data reported in this paper have been deposited in the Genome Sequence Archive in National Genomics Data Center, China National Center for Bioinformation/Beijing Institute of Genomics, Chinese Academy of Sciences (GSA: CRA027295) that were publicly accessible at https://ngdc.cncb.ac.cn/gsa.

### Molecular Docking

The 3D structures of GLS1, POLR2E, and POLR2E were obtained from the protein database (PDB:3VOY for GLS1, and PDB:7OB9 for POLR2E/POLR2H). Docking calculations of GLS1 with POLR2E or POLR2H were carried out using ZDOCK 3.0.2 webserver following IRaPPA re‐ranking.^[^ ^38^ ^]^ The active binding sites for protein‐protein docking were selected based on the experiment results. The protein complex with the lowest binding energy was used to perform the molecular dynamics (MD) simulations.

The protein complex systems performed all‐atom MD simulations on the GROMACS platform with high efficiency by GPU acceleration.^[^ ^39^ ^]^ Each system was placed into a 0.15 M NaCl water box, the smallest distance between the box boundary and the biomolecule was 15 Å. The periodic boundary conditions (PBC) were employed on all three dimensions. The Amber ff14SB force fields were used to describe the protein.^[^ ^40^ ^]^ The TIP3P model was chosen to reproduce the water. For each simulation system, energy minimization was performed (5000 steepest descent steps, followed by 5000 conjugated gradient algorithm steps). The minimized structure was first gradually heated to 300 K for 0.5 ns and equilibrated for 2 ns. Finally, 500 ns long productive MD simulations in an NPT ensemble (300 K and 1 bar) were carried out. Details of the simulation parameters could be referred to in the previous work.^[^ ^41^ ^]^ In the present study, hydrogen bonds were defined to be present if the distance between the acceptor and donor atoms was below 3.5 Å and the angle among the hydrogen‐donor‐acceptor atoms was below 30°. The salt bridge interactions were defined as the distance between two charged groups smaller than 5 Å. The binding free energy profiles of GLS1 and POLR2E/POLR2H were calculated via the gmx_MMPBSA (v1.6.2) tool, which utilized the Molecular Mechanics Poisson‐Boltzmann Surface Area (MMPBSA) algorithm.^[^ ^42^ ^]^

### Transcriptomic Analysis

Total RNA was isolated from liver samples of mice using Trizol (15596018; Life Technologies) with phase separation. RNA was submitted to Majorbio (Shanghai, China) for RNA‐Seq transcriptome sequencing and data analyzing using the BGISEQ‐500 platform. Pathway enrichment analysis approaches analyze a ranked gene list filtered by a particular threshold (FDR‐adjusted P value <0.05, fold change >2). All sequencing data reported in this paper have been deposited in the Genome Sequence Archive in National Genomics Data Center, China National Center for Bioinformation / Beijing Institute of Genomics, Chinese Academy of Sciences (GSA: CRA014391) that were publicly accessible at https://ngdc.cncb.ac.cn/gsa.

### Statistical Analysis

Normalized data on cellular triglyceride (TG) content, mRNA levels, luciferase activity, EU‐click assays and bodipy staining intensity. All data were expressed as the mean ± SEM and were statistically analyzed using GraphPad Prism v8.0 (GraphPad Software Inc, USA). Differences between two groups were analyzed by unpaired two‐sided Student's *t*‐test. Statistical analysis for multiple groups was performed by one‐way ANOVA or two‐way ANOVA and Fisher's LSD test. A P value of ≤0.05 was considered significant.

## Conflict of Interest

The authors declare no conflict of interest.

## Author Contributions

W.W., H.J., Y.L. contributed equally to this work and share the first authorship. WBW and HWJ conceived the projected and wrote the manuscript. WBW completed primary experiments. YCL and JLL engage in the evaluation of molecular docking. LYJ and CP helped construct the plasmid. HLW supported data processing for RNA sequencing and analysis of database data. ZL, WHY, HM, HYG and YS contributed to animal experiments. SFL, RW, JL and HWJ conceived of the study and supervised the work.

## Supporting information



Supporting Information

Supplemental DataFile 1

## Data Availability

The data that support the findings of this study are available from the corresponding author upon reasonable request.
