# Supplementary material for: GLS1‐RNA Polymerase II Axis Mediates Glutamine‐Dependent Hepatoprotective Effects on Alcoholic Liver Disease in High‐Protein Diets
Source: Adv Sci (Weinh). 2025 Sep 11;12(45):e02738. doi: 10.1002/advs.202502738 (PMC12677690; doi:10.1002/advs.202502738)
Supplement: Supplementary file 1 — Supporting Information [file ADVS-12-e02738-s002.pdf]

Supplemental information

GLS1-RNA Polymerase II Axis Mediates Glutamine-Dependent Hepatoprotective Effects on Alcoholic Liver Disease in High-Protein Diets

Wenbiao Wu, Haowen Jiang, Yichang Liu, Chang Peng, Hanlin Wang, Wenhua Yang, Zan Lyu, Yan Sun, Huan Ma, Hongyu Gu, Weijuan Kan, Liya Jing, Tiancheng Dong, Chunmei Xia, Saifei Lei, Rui Wu, Jinlong Li, Jia Li

GLS1-RNA Polymerase II Axis Mediates Glutamine-Dependent Hepatoprotective Effects on Alcoholic Liver Disease in High-Protein Diets

Wenbiao Wu, Haowen Jiang, Yichang Liu, Chang Peng, Hanlin Wang, Wenhua Yang, Zan Lyu, Yan Sun, Huan Ma, Hongyu Gu, Weijuan Kan, Liya Jing, Tiancheng Dong, Chunmei Xia, Saifei Lei, Rui Wu, Jinlong Li, Jia Li

Table of contents

Supplementary Figures……………………………………………..……....3

Supplementary Tables…………………………………………………………24

Supplementary Figures

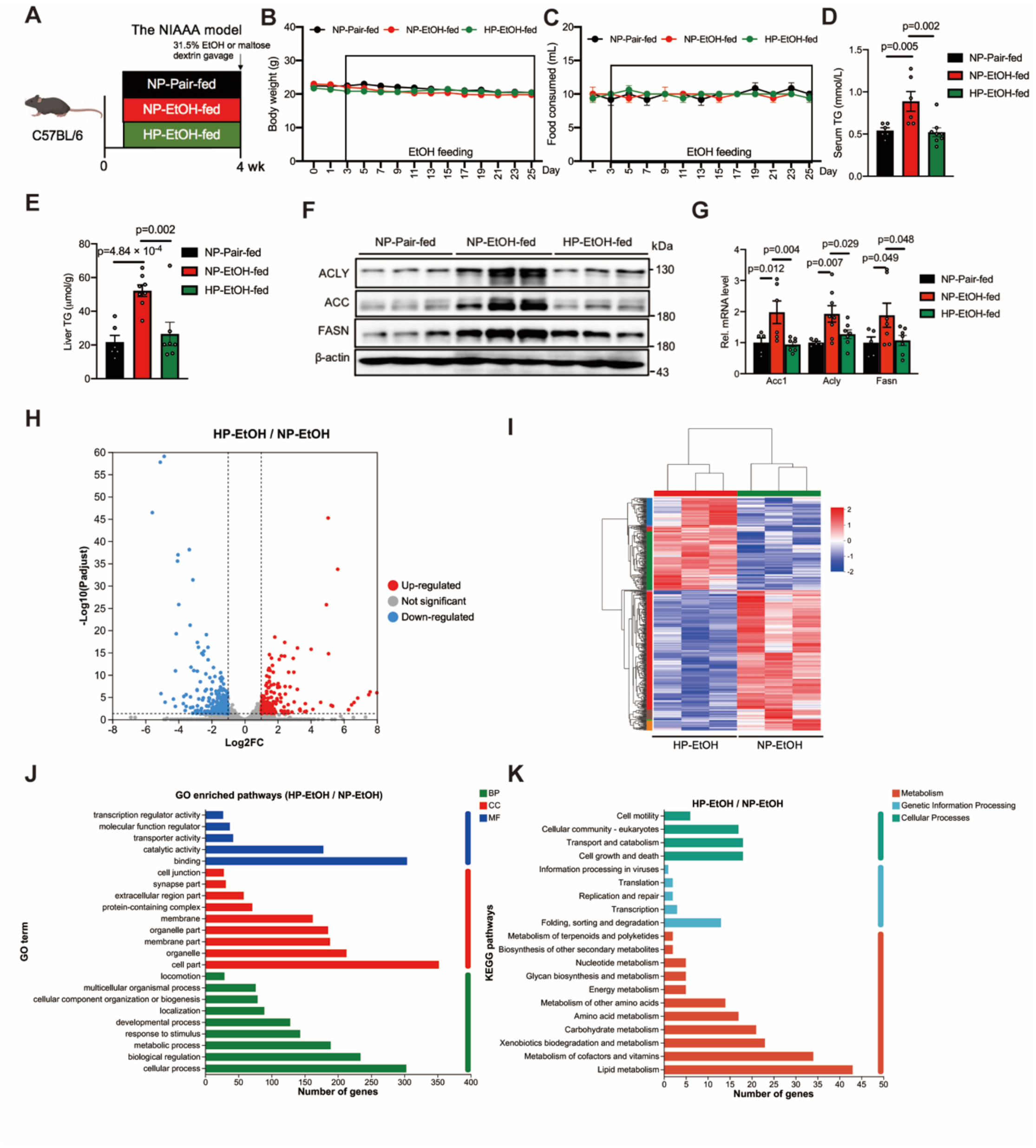

Figure S1. High protein diet attenuates alcoholic hepatic steatosis.

(A) Schematic illustrating the groups and procedures of the mouse model. C57BL/6J mice were fed with normal-protein diet or high-protein diet for 4 weeks. n=6-8 biologically independent mice per group.

(B-C) Change curves of body weight, food intake of mice.

(D-E) Serum TG and liver TG levels in the indicated group in (A); n = 6-8.

(F) Western blots of ACLY, ACC, FASN levels in liver of the mice described in (A); β-actin served as the loading control.

(G) qPCR of Acly, Acc1, Fasn mRNA levels in liver of the mice described in (A); β-actin served as the control. n=4-8.

(H) A volcano plot showing differentially expressed genes in HP-EtOH vs. NP- EtOH groups. Red, upregulated genes; blue, downregulated genes, gray, no significant change.

(I) Heat map of differentially expressed genes in HP-EtOH vs. NP-EtOH groups. n = 3.

(J) GO enrichment analysis of differentially expressed genes in HP-EtOH vs. NP-EtOH groups.

(K) KEGG enrichment analysis of differentially expressed genes in HP-EtOH vs. NP-EtOH groups.

Data in (D-E) and (G) are presented as the mean ± SEM, determined by one-way ANOVA and Fisher's LSD test.

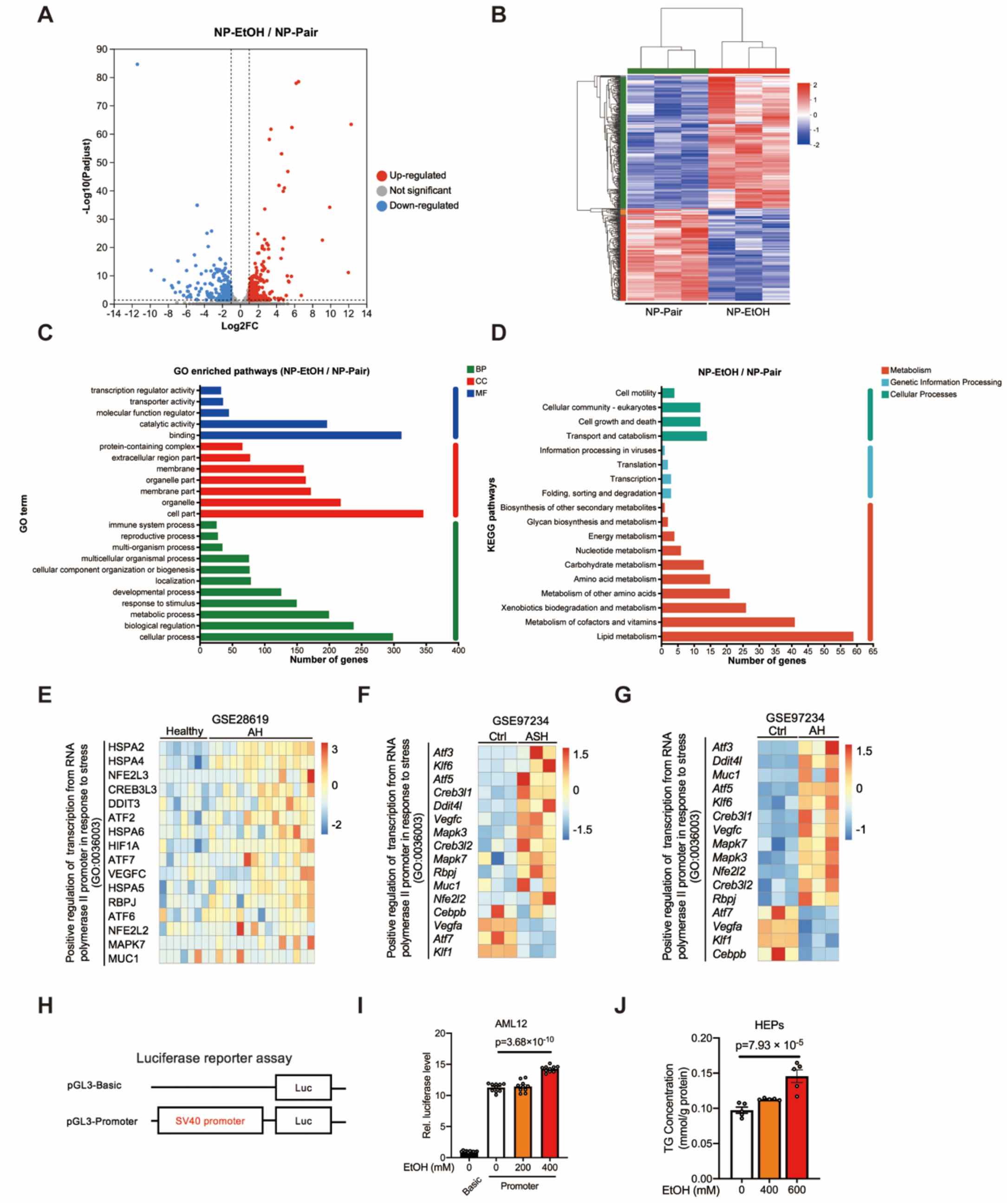

Figure S2. Alcoholic hepatic steatosis is associated with RNA pol II activity.

(A) A volcano plot showing differentially expressed genes in NP-EtOH vs. NP-Pair groups. Red, upregulated genes; blue, downregulated genes, gray, no significant change.

(B) Heat map of differentially expressed genes in NP-EtOH vs. NP-Pair groups. n = 3.

(C) GO enrichment analysis of differentially expressed genes in NP-EtOH vs. NP-Pair groups.

(D) KEGG enrichment analysis of differentially expressed genes in NP-EtOH vs. NP-Pair groups.

(E) Heatmaps representation of genes annotated by positive regulation of transcription from RNA polymerase II promoter in response to stress (GO: 0036003) in healthy controls and patients with AH (GEO accession number: GSE28619).

(F-G) RNA sequencing data (GEO accession number: GSE97234) of mouse livers with control (n = 3), alcoholic steatohepatitis (ASH, n = 3) and alcoholic hepatitis (AH, n = 3). Heatmaps representation of genes annotated by positive regulation of transcription from RNA polymerase II promoter in response to stress (GO: 0036003) in control vs ASH or AH.

(H) Schematic diagram illustrating the building strategy of luciferase reporter assay.

(I) Luciferase levels of AML12 cells expressing pGL3-Basic or pGL3-Promoter. Cells were serum starved overnight followed by ethanol (200 mM and 400 mM) stimulation for 1 hr. n=10.

(J) TG levels of the primary hepatocytes treated with ethanol (400, 600 mM) for 24 hr. The amount was normalized to the protein content. n=5.

Data in (I-J) are presented as the mean ± SEM, determined by one-way ANOVA and Fisher's LSD test.

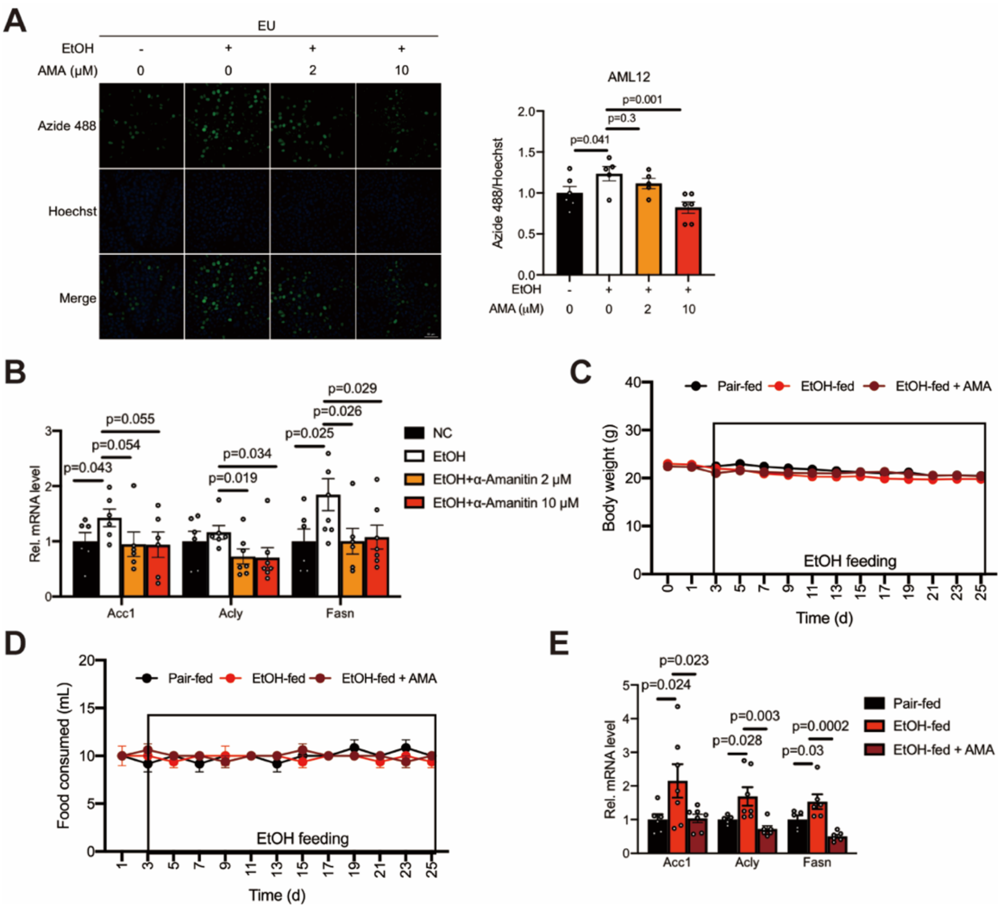

Figure S3. AMA attenuates alcoholic hepatic steatosis.

(A) Overall transcription levels of AML12 cells treated with AMA (2, 10 μM) and ethanol (400 mM) for 12 hr using EU-click assays. The amount was normalized to the hoechst content. n=5-6. Scale bars, 50 μm.

(B) mRNA levels of the primary hepatocytes of C57BL/6J mice treated with α-Amanitin (2, 10 μM) and ethanol (600 mM) for 24 hr. β-actin served as the control. n=6-8.

(C-D) Change curves of body weight, food intake of mice injected with vehicle or α-Amanitin.

(E) qPCR of Acly, Acc1, Fasn mRNA levels in liver of the mice described in (C); β-actin served as the control. n=5-7.

Data in (A-B) and (E) are presented as the mean ± SEM, determined by one-way ANOVA and Fisher's LSD test.

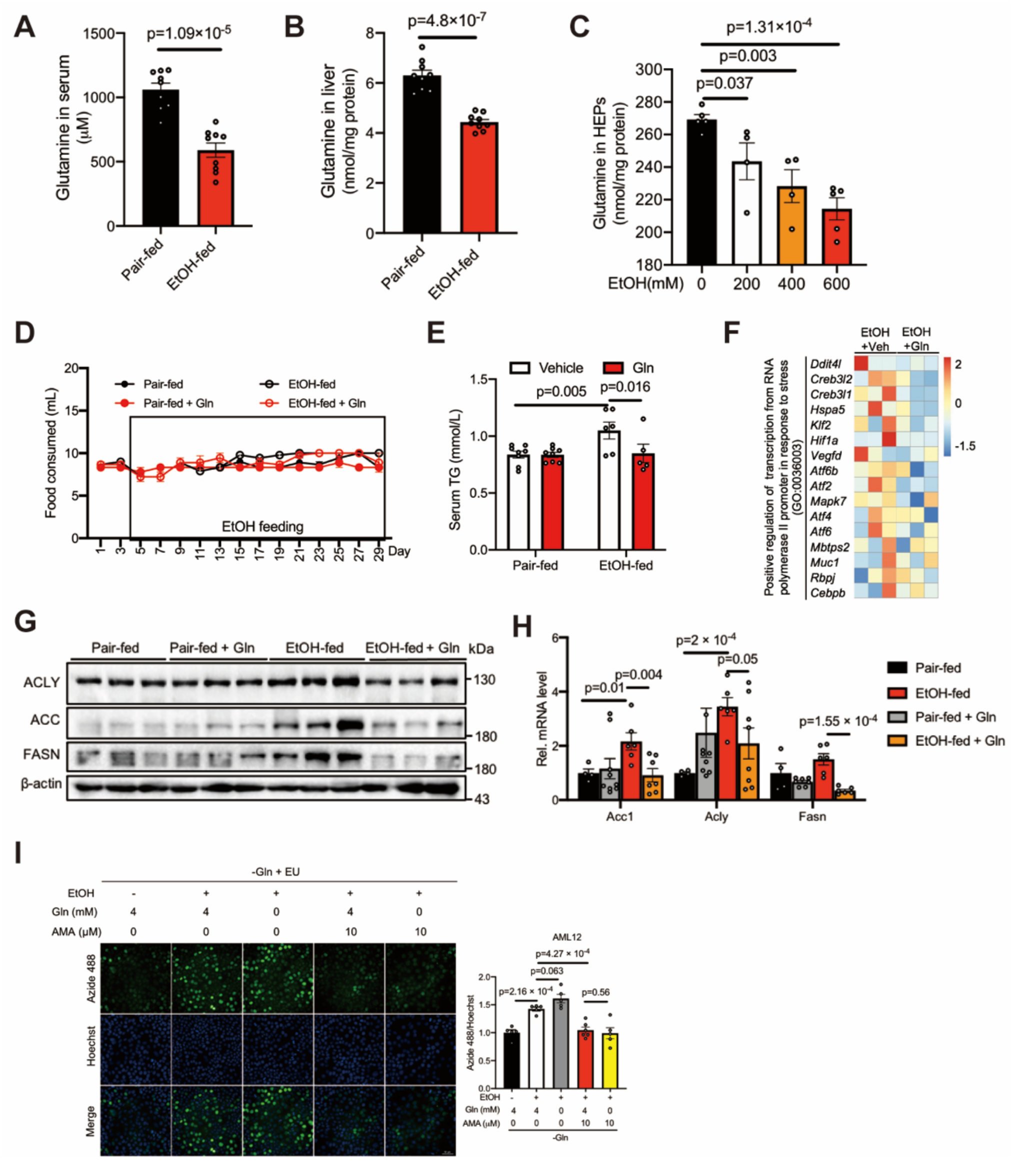

Figure S4. Glutamine attenuates alcoholic hepatic steatosis.

(A) Glutamine concentration in the mice serum after 4 weeks in the EtOH-induced NIAAA ALD model and control mice. n = 9.

(B) Glutamine concentration in the mice liver after 4 weeks in the EtOH-induced NIAAA ALD model and control mice. n = 9.

(C) Glutamine concentration in the primary hepatocytes treated with ethanol (200, 400, 600 mM) for 24 hr. n=4-5.

(D) Change curves of food intake of mice fed with glutamine.

(E) Serum TG levels in mouse model described in (D); n = 5-8.

(F) Heatmaps representation of genes annotated by positive regulation of transcription from RNA polymerase II promoter in response to stress (GO: 0036003) in EtOH-fed group and EtOH-fed with glutamine group.

(G) Western blots of ACLY, ACC, FASN levels in liver of the mice described in (D); β-actin served as the loading control.

(H) qPCR of Acly, Acc1, Fasn mRNA levels in liver of the mice described in (D); β-actin served as the control. n=4-9.

(I) Overall transcription levels of AML12 cells treated with AMA (10 μM) or glutamine (4 mM) and ethanol (400 mM) in glutamine-free medium for 12hr using EU-click assays. The amount was normalized to the hoechst content. n=5-6. Scale bars, 50 μm.

Data in (A) and (B) are presented as the mean ± SEM, determined by unpaired two-sided Student's t-test. Data in (C) and (I) are presented as the mean ± SEM, determined by one-way ANOVA and Fisher's LSD test. Data in (E) and (H) are presented as the mean ± SEM, determined by two-way ANOVA and Fisher's LSD test.

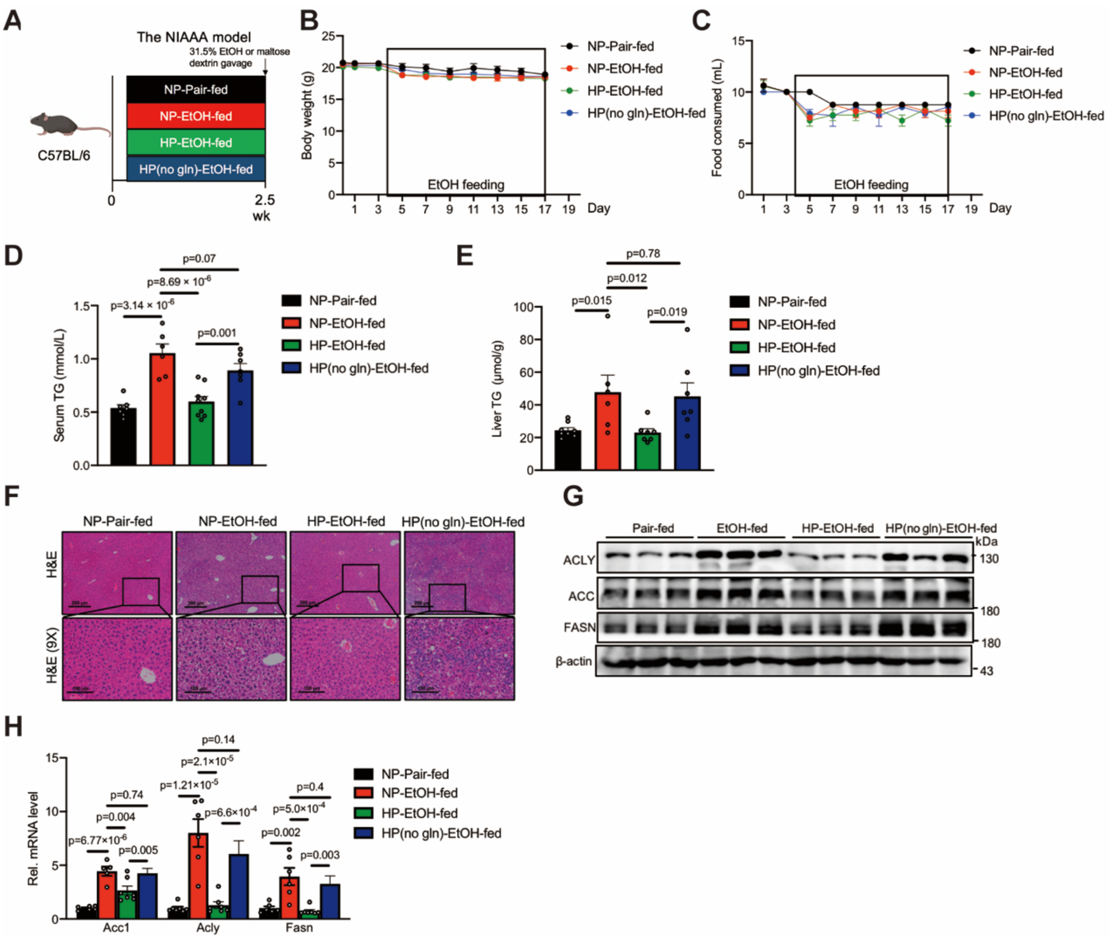

Figure S5. Glutamine in a high-protein diet plays a key role in attenuating alcoholic hepatic steatosis.

(A) Schematic illustrating the groups and procedures of the normal-protein diet, high-protein diet and glutamine-deficient high-protein diet AFLD model. n=6-9 biologically independent mice per group.

(B-C) Change curves of body weight, food intake of mice in the indicated group from (A).

(D-E) Serum TG and liver TG levels in the indicated group from (A); n = 6-9.

(F) Representative H&E staining of liver sections in the indicated group in (A). Scale bars, 100 μm.

(G) Western blots of ACLY, ACC, FASN levels in liver of the mice described in (A); β-actin served as the loading control.

(H) qPCR of Acly, Acc1, Fasn mRNA levels in liver of the mice described in (A); β-actin served as the control. n=5-7.

Data in (D-E) and (H) are presented as the mean ± SEM, determined by one-way ANOVA and Fisher's LSD test.

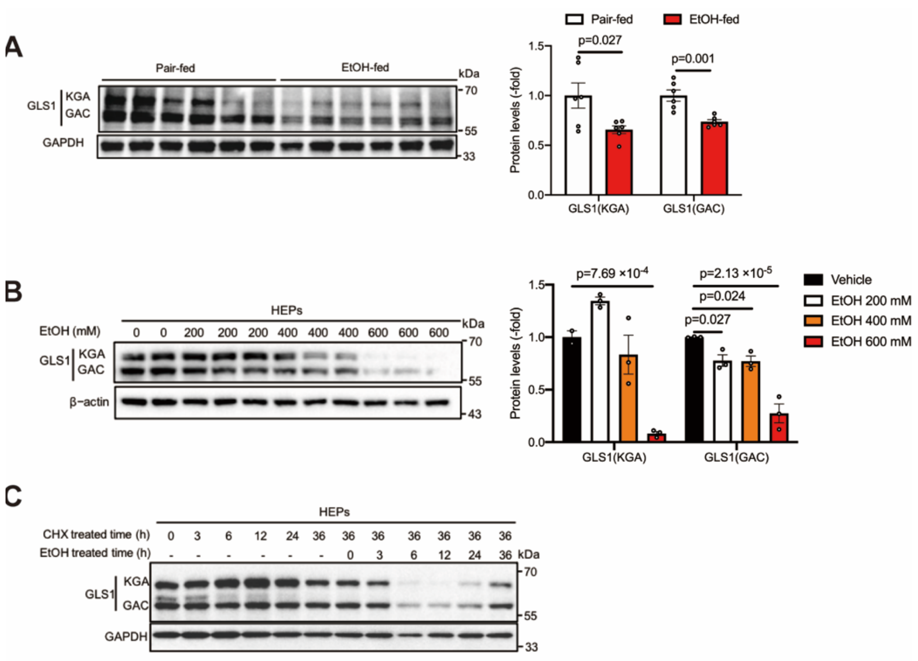

Figure S6. Hepatic GLS1 protein is downregulated in response to ethanol.

(A) Western blots of hepatic GLS1 after 4 weeks in the EtOH-induced NIAAA ALD model and control mice. n = 6.

(B) Western blots of GLS1 levels in the primary hepatocytes treated with ethanol (200, 400, 600 mM) for 24 hr. β-actin serves as a loading control. n=2-3.

(C) Western blots of GLS1 levels in the primary hepatocytes treated with ethanol (600 mM) for 3, 6, 12, 24, 36 h and CHX (Cycloheximide, 10 μg/ml) for 36 hr. GAPDH serves as a loading control.

Data in (A-B) are presented as the mean ± SEM, determined by unpaired two-sided Student's t-test.

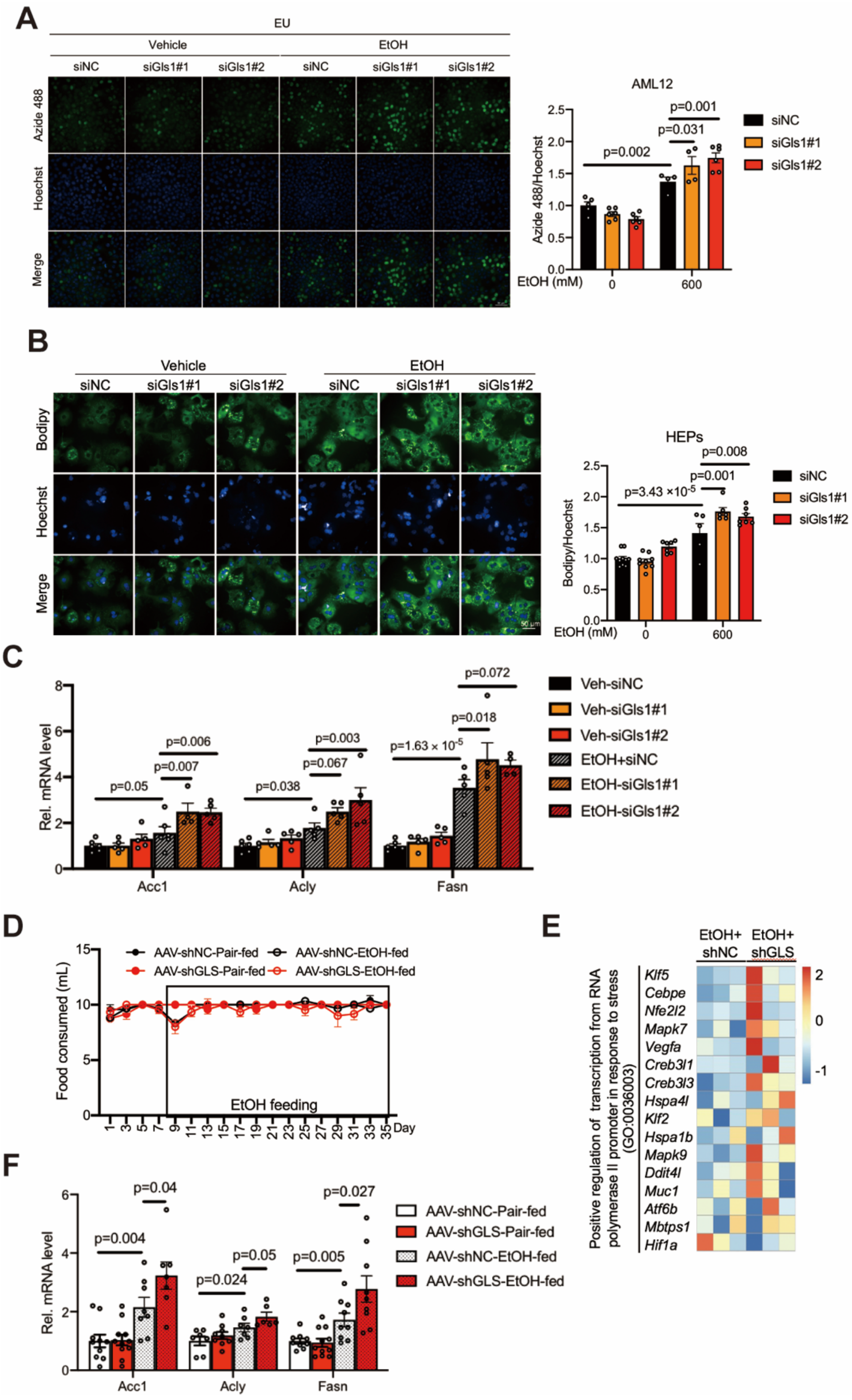

Figure S7. Intervention of *Gls1* increases liver triacylglycerol deposition induced by ethanol.

(A) Overall transcription levels of AML12 cells transfected with siRNAs targeting GLS1 or control using EU-click assays. Cells were serum starved overnight followed

by ethanol (400 mM) stimulation for 12 hr. The amount was normalized to the hoechst content. n=4-6. Scale bars, 50 μm.

(B) Bodipy levels of the primary hepatocytes transfected with siRNAs targeting GLS1 or control. Cells were serum starved overnight followed by ethanol (600 mM) stimulation for 24 hr. Scale bars, 50 μm. The amount was normalized to the hoechst content. n=5-10.

(C) mRNA levels of the primary hepatocytes transfected with siRNAs targeting GLS1 or control. Cells were serum starved overnight followed by ethanol (600 mM) stimulation for 24 hr. β-actin serves as the control. n=4-6.

(D) Change curves of food intake of mice in the AAV-shGLS group or AAV-shNC group.

(E) Heatmaps representation of genes annotated by positive regulation of transcription from RNA polymerase II promoter in response to stress (GO: 0036003) in the AAV-shGLS group or AAV-shNC group.

(F) qPCR of Acly, Acc1, Fasn mRNA levels in liver of the mice described in (D); β-actin served as the control. n=6-10.

Data in (A-C) and (F) are presented as the mean ± SEM, determined by two-way ANOVA and Fisher's LSD test.

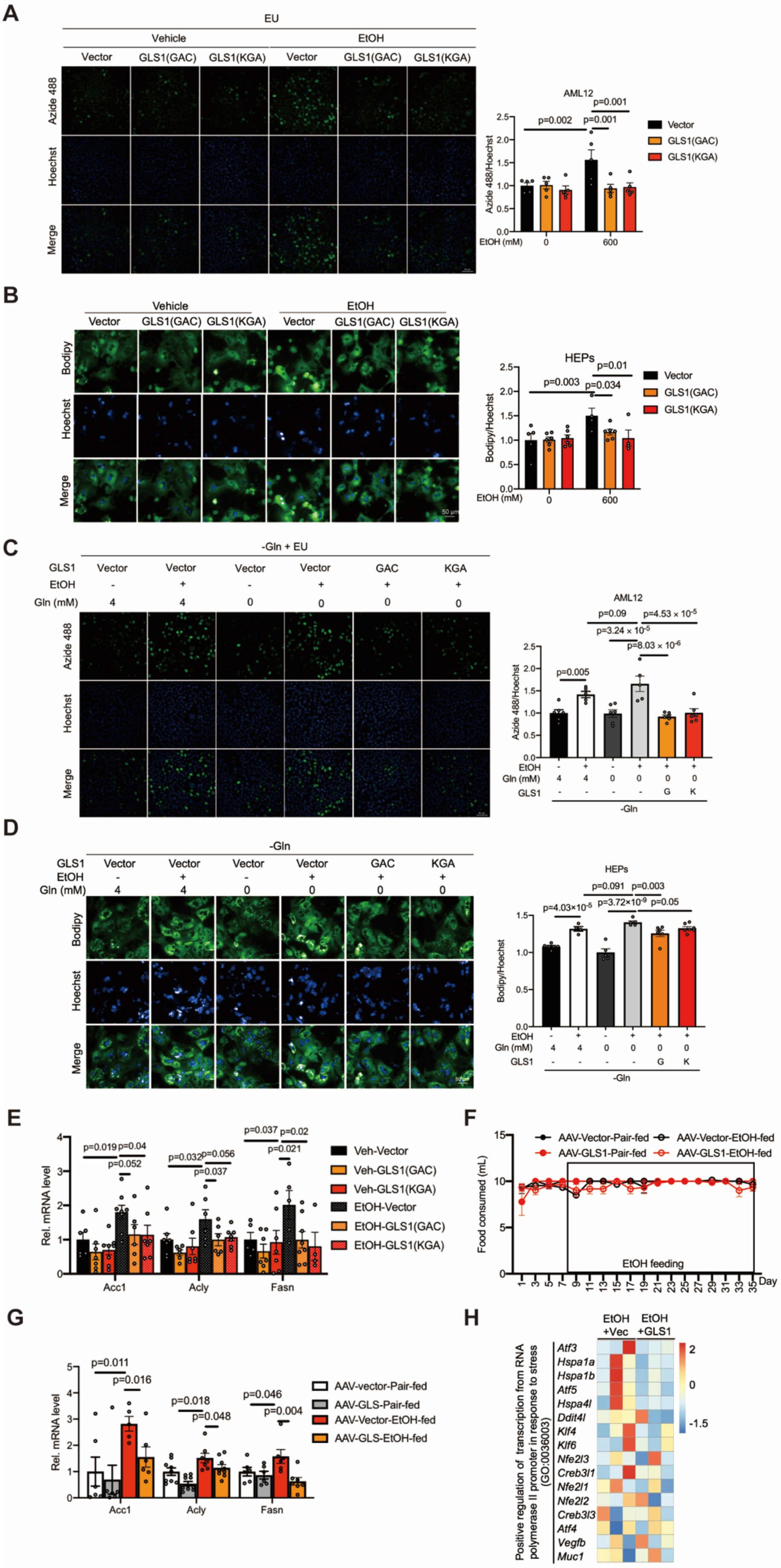
A
EU
Vehicle
EtOH
Vector
GLS1(GAC)
GLS1(KGA)
Azide 488
Hoechst
Merge
AML12
Azide 488/Hoechst
EtOH (mM)
B
Bodipy
HEPs
Bodipy/Hoechst
C
-Gln + EU
GLS1
EtOH
Gln (mM)
-Gln
D
-Gln
E
Rel. mRNA level
Veh-Vector
Veh-GLS1(GAC)
Veh-GLS1(KGA)
EtOH-Vector
EtOH-GLS1(GAC)
EtOH-GLS1(KGA)
Acc1
Acly
Fasn
F
AAV-Vector-Pair-fed
AAV-Vector-EtOH-fed
AAV-GLS1-Pair-fed
AAV-GLS1-EtOH-fed
Food consumed (mL)
EtOH feeding
Day
G
AAV-vector-Pair-fed
AAV-GLS-Pair-fed
AAV-Vector-EtOH-fed
AAV-GLS-EtOH-fed
H
EtOH +Vec
EtOH +GLS1
Positive regulation of transcription from RNA polymerase II promoter in response to stress (GO:0036003)
Atf3
Hspa1a
Hspa1b
Atf5
Hspa4l
Ddit4l
Klf4
Klf6
Nfe2l3
Creb3l1
Nfe2l1
Nfe2l2
Creb3l3
Atf4
Vegfb
Muc1

Figure S8. GLS1 reduces excessive liver triacylglycerol deposition induced by ethanol. (A) Overall transcription levels of AML12 cells expressing GLS1 using EU-click assays. Cells were serum starved overnight followed by ethanol (400 mM) stimulation for 12 hr. The amount was normalized to the hoechst content. n=5. Scale bars, 50 μm.
(B) Bodipy levels of the primary hepatocytes expressing GLS1. Cells were serum starved overnight followed by ethanol (600 mM) stimulation for 24 hr. Scale bars, 50 μm. The amount was normalized to the hoechst content. n=4-6.
(C) Overall transcription levels of AML12 cells expressing GLS1 using EU-click assays. Cells were serum starved overnight in glutamine-free medium followed by ethanol (400 mM) stimulation for 12 hr. The amount was normalized to the hoechst content. n=5-6. Scale bars, 50 μm.
(D) Bodipy levels of the primary hepatocytes expressing GLS1. Cells were serum starved overnight in glutamine-free medium followed by ethanol (600 mM) stimulation for 24 hr. Scale bars, 50 μm. The amount was normalized to the hoechst content. n=4-7.
(E) mRNA levels of the primary hepatocytes described in (B). β-actin serves as the control. n=4-8.
(F) Change curves of food intake of mice in the AAV-GLS1 group or AAV-Vector group.
(G) qPCR of Acly, Acc1, Fasn mRNA levels in liver of the mice described in (F); β-actin served as the control. n=6-9.
(H) Heatmaps representation of genes annotated by positive regulation of transcription from RNA polymerase II promoter in response to stress (GO: 0036003) in the AAV-GLS1 group or AAV-Vector group.
Data in (A-B), (E) and (G) are presented as the mean ± SEM, determined by two-way ANOVA and Fisher's LSD test. Data in (C-D) are presented as the mean ± SEM, determined by one-way ANOVA and Fisher's LSD test.

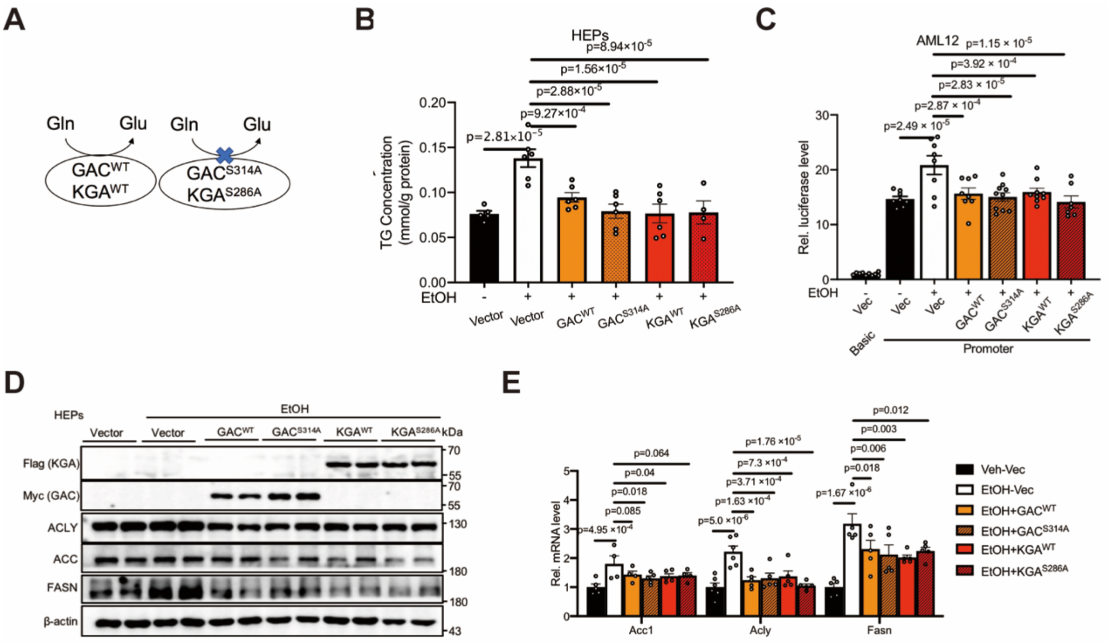

Figure S9. Catalytically inactive GLS1 mutants reduce excessive liver triacylglycerol deposition induced by ethanol.

(A) Schematic diagram of the GAC S314A and KGA S286A mutant not exerting metabolic enzyme activity.

(B) TG levels of the primary hepatocytes expressing with GAC WT or GAC S314A or KGA WT or KGA S286A. Cells were serum starved overnight followed by ethanol (600 mM) stimulation for 24 hr. The amount was normalized to the protein content. n=4-6.

(C) RNA pol II activity of AML12 cells expressing pGL3-Basic or pGL3-Promoter with GAC WT or GAC S314A or KGA WT or KGA S286A. Cells were serum starved overnight followed by ethanol (400 mM) stimulation for 1 hr. n=8-10.

(D) Western blots of ACLY, ACC, FASN levels in the primary hepatocytes described in (B); β-actin serves as a loading control.

(E) mRNA levels of the primary hepatocytes described in (B). β-actin serves as the control. n=4-6.

Data in (B-C) and (E) are presented as the mean ± SEM, determined by one-way ANOVA and Fisher's LSD test.

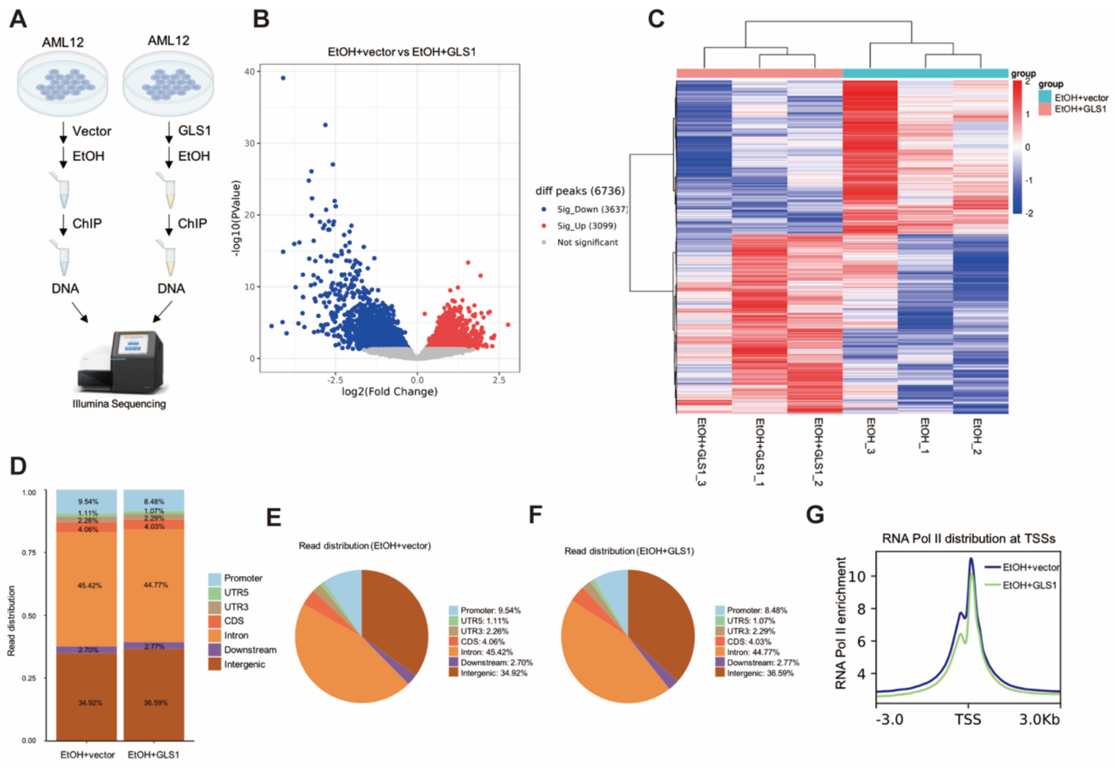

Figure S10. GLS1 influences RNA pol II activity at transcription initiation.

(A) Schematic of ChIP-seq to elucidate the mechanism of GLS1-regulated RNA pol II activity in AML12 cells. n = 3 biologically independent samples per condition.

(B) A volcano plot showing differentially expressed genes in EtOH vs. EtOH+GLS1 groups. Red, upregulated genes; blue, downregulated genes, gray, no significant change. n = 3 biologically independent samples per condition.

(C) Heat map of differentially expressed genes in EtOH vs. EtOH+GLS1 groups. n = 3 biologically independent samples per condition.

(D-F) RNA pol II binding peaks in EtOH treated cells or GLS1-overexpressed EtOH treated cells were categorized into promoter region (blue), 5’UTR region (green), 3’UTR region (gray), CDS region (red), intron region (orange), downstream region (purple), and intergenic region (brown). n = 3 biologically independent samples per condition.

(G) RNA pol II metagene line plots showing read enrichment around TSSs (±75 bp) of all mouse genes in EtOH and EtOH+GLS1 groups: EtOH (blue) and EtOH+GLS1 (green). n = 3 biologically independent samples per condition.

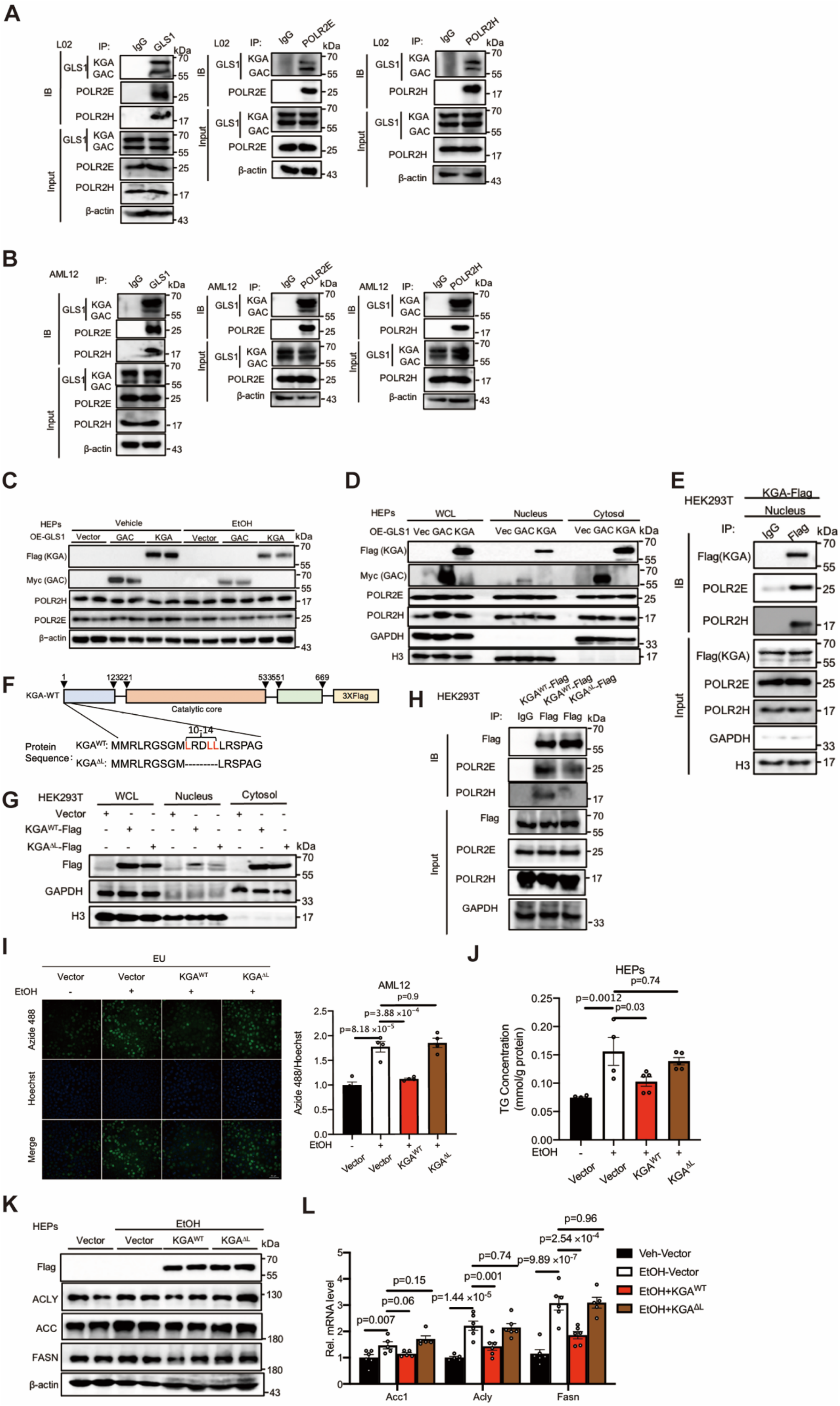

A
B
C
D
E
F
G
H
I
J
K
L

Figure S11. GLS1 interacts with POLR2E or POLR2H in nucleus.

(A) Co-IP analysis of the interaction of GLS1 with POLR2E or POLR2H in the LO2 cells. β-actin served as the loading control.

(B) Co-IP analysis of the interaction of GLS1 with POLR2E or POLR2H in the AML12 cells. β-actin served as the loading control.

(C) Western blots of POLR2H, POLR2E levels in the primary hepatocytes expressing GLS1. Cells were serum starved overnight followed by ethanol (600 mM) stimulation for 24 hr. β-actin serves as a loading control.

(D) Western blots of KGA-Flag, GAC-Myc, and endogenous POLR2H, POLR2E levels in the primary hepatocytes expressing GLS1 after nuclear and cytoplasmic extraction. Histone 3 and GAPDH served as a loading control for nucleus and cytoplasmic fractions, respectively.

(E) Co-IP analysis of the interaction of KGA-Flag with endogenous POLR2H or POLR2E after nuclear and cytoplasmic extraction in HEK293T cells. Histone 3 and GAPDH served as a loading control for nucleus and cytoplasmic fractions, respectively.

(F) Schematic diagram of the KGA ΔL mutant.

(G) Western blots of KGA-WT-Flag and KGA-ΔL-Flag levels in HEK293T cells after nuclear and cytoplasmic extraction. Histone 3 and GAPDH served as a loading control for nucleus and cytoplasmic fractions, respectively.

(H) Co-IP analysis of the interaction of KGA-WT-Flag and KGA-ΔL-Flag with endogenous POLR2H or POLR2E in HEK293T cells. GAPDH served as a loading control.

(I) Overall transcription levels of AML12 cells expressing with KGA-WT-Flag and KGA-ΔL-Flag using EU-click assays. Cells were serum starved overnight followed by ethanol (400 mM) stimulation for 12 hr. The amount was normalized to the hoechst content. n=4. Scale bars, 50 μm.

(J) TG levels of the primary hepatocytes expressing with KGA-WT-Flag and KGA-ΔL-Flag. Cells were serum starved overnight followed by ethanol (600 mM) stimulation for 24 hr. The amount was normalized to the protein content. n=4-5.

(K) Western blots of ACLY, ACC, FASN levels in the primary hepatocytes described in

(J); β-actin serves as a loading control.

(L) mRNA levels of the primary hepatocytes described in (J). β-actin serves as the control. n=5-6.

Data in (I-J) and (L) are presented as the mean ± SEM, determined by one-way ANOVA and Fisher's LSD test.

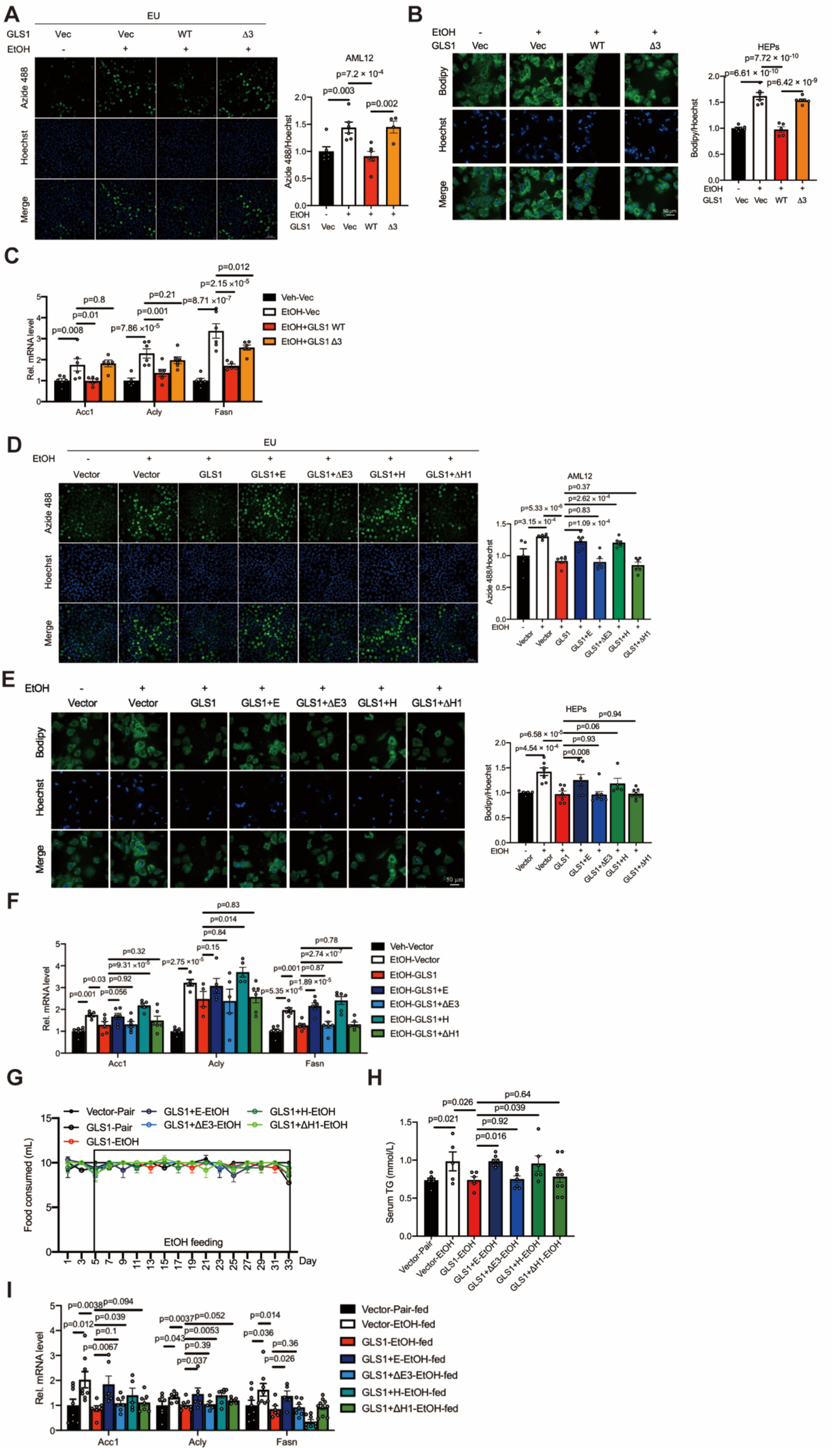
A
EU
GLS1 Vec Vec WT Δ3
EtOH - + + +
Azide 488
Hoechst
Merge
AML12
p=7.2 × 10-4
p=0.003
p=0.002
Azide 488/Hoechst
B
EtOH - + + +
GLS1 Vec Vec WT Δ3
Bodipy
Hoechst
Merge
HEPs
p=7.72 × 10-10
p=6.61 × 10-10
p=6.42 × 10-9
Bodipy/Hoechst
C
p=0.012
p=2.15 ×10-5
p=0.8
p=0.21
p=8.71 ×10-7
p=0.01
p=0.001
p=0.008
p=7.86 ×10-5
Rel. mRNA level
Acc1
Acly
Fasn
Veh-Vec
EtOH-Vec
EtOH+GLS1 WT
EtOH+GLS1 Δ3
D
EU
EtOH - + + + + + +
Vector Vector GLS1 GLS1+E GLS1+ΔE3 GLS1+H GLS1+ΔH1
Azide 488
Hoechst
Merge
AML12
p=0.37
p=2.62 × 10-4
p=5.33 × 10-4
p=0.83
p=3.15 × 10-4
p=1.09 × 10-4
Azide 488/Hoechst
E
EtOH - + + + + + +
Vector Vector GLS1 GLS1+E GLS1+ΔE3 GLS1+H GLS1+ΔH1
Bodipy
Hoechst
Merge
50 μm
HEPs
p=0.94
p=0.06
p=6.58 × 10-5
p=0.93
p=4.54 × 10-4
p=0.008
Bodipy/Hoechst
F
p=0.83
p=0.014
p=0.84
p=0.15
p=0.78
p=0.32
p=2.74 ×10-7
p=9.31 ×10-5
p=2.75 ×10-5
p=0.001
p=0.87
p=0.03
p=0.92
p=1.89 ×10-5
p=0.001
p=0.056
p=5.35 ×10-6
Rel. mRNA level
Acc1
Acly
Fasn
Veh-Vector
EtOH-Vector
EtOH-GLS1
EtOH-GLS1+E
EtOH-GLS1+ΔE3
EtOH-GLS1+H
EtOH-GLS1+ΔH1
G
Vector-Pair
GLS1-Pair
GLS1-EtOH
GLS1+E-EtOH
GLS1+ΔE3-EtOH
GLS1+H-EtOH
GLS1+ΔH1-EtOH
Food consumed (mL)
EtOH feeding
Day
H
p=0.64
p=0.026
p=0.039
p=0.021
p=0.92
p=0.016
Serum TG (mmol/L)
Vector-Pair
Vector-EtOH
GLS1-EtOH
GLS1+E-EtOH
GLS1+ΔE3-EtOH
GLS1+H-EtOH
GLS1+ΔH1-EtOH
I
p=0.0038
p=0.094
p=0.039
p=0.012
p=0.1
p=0.0067
p=0.0037
p=0.052
p=0.0053
p=0.043
p=0.39
p=0.037
p=0.014
p=0.036
p=0.36
p=0.026
Rel. mRNA level
Acc1
Acly
Fasn
Vector-Pair-fed
Vector-EtOH-fed
GLS1-EtOH-fed
GLS1+E-EtOH-fed
GLS1+ΔE3-EtOH-fed
GLS1+H-EtOH-fed
GLS1+ΔH1-EtOH-fed

Figure S12. GLS1 reduces triacylglycerol deposition induced by ethanol through interacting with POLR2E or POLR2H.

(A) Overall transcription levels of AML12 cells expressing with KGA WT or ΔKGA-3 using EU-click assays. Cells were serum starved overnight followed by ethanol (400 mM) stimulation for 12 hr. The amount was normalized to the hoechst content. n=4-6. Scale bars, 50 μm.

(B) Bodipy levels of the primary hepatocytes expressing with KGA WT or ΔKGA-3. Cells were serum starved overnight followed by ethanol (600 mM) stimulation for 24 hr. Scale bars, 50 μm. The amount was normalized to the hoechst content. n=5-6.

(C) mRNA levels of the primary hepatocytes described in (B). β-actin serves as the control. n=5-6.

(D) Overall transcription levels of AML12 cells expressing with GLS1 and POLR2E WT or ΔPOLR2E-3 or POLR2H WT or ΔPOLR2H-1 using EU-click assays. Cells were serum starved overnight followed by ethanol (400 mM) stimulation for 12 hr. The amount was normalized to the hoechst content. n=5-6. Scale bars, 50 μm.

(E) Bodipy levels of the primary hepatocytes expressing with GLS1 and POLR2E WT or ΔPOLR2E-3 or POLR2H WT or ΔPOLR2H-1. Cells were serum starved overnight followed by ethanol (600 mM) stimulation for 24 hr. Scale bars, 50 μm. The amount was normalized to the hoechst content. n=5-8.

(F) mRNA levels of the primary hepatocytes described in (E). β-actin serves as the control. n=4-6.

(G) Change curves of food intake of mice in the GLS1 with POLR2E WT or POLR2H WT or their truncated variants (ΔPOLR2E-3 or ΔPOLR2H-1) groups.

(H) Serum TG levels of mice in the indicated group in (G); n = 5-9.

(I) qPCR of Acly, Acc1, Fasn mRNA levels in liver of the mice described in (G); β-actin served as the control. n=5-10.

Data in (A-F), (H-I) are presented as the mean ± SEM, determined by one-way ANOVA and Fisher's LSD test.

Supplementary Table

Table S1. Ingredients of diets used in this study

Composition of different diets										
			NP-Pair		NP-EtOH		HP-EtOH		HP(no gln)-EtOH	
Class	Ingredient	kcal/g	gram	kcal	gram	kcal	gram	kcal	gram	kcal
Protein	Caisein	4	41.7	167	41.7	167	84.5	338	0	0
AA	L-Cystine	4	0.5	2	0.5	2	0.3	1.2	2	8
	L-Alanine	4	0	0	0	0	0	0	5	20
	L-Arginine	4	0	0	0	0	0	0	5	20
	L-Asparagine	4	0	0	0	0	0	0	2.5	10
	L-Aspartate	4	0	0	0	0	0	0	5	20
	L-Glutamic Acid	4	0	0	0	0	0	0	17.5	70
	L-Glutamine	4	0	0	0	0	0	0	0	0
	Glycine	4	0	0	0	0	0	0	5	20
	L-Proline	4	0	0	0	0	0	0	2.5	10
	L-Serine	4	0	0	0	0	0	0	2.5	20
	L-Tyrosine	4	0	0	0	0	0	0	2	8
EAA	L-Histidine	4	0	0	0	0	0	0	3	12
	L-Isoleucine	4	0	0	0	0	0	0	4	16
	L-Leucine	4	0	0	0	0	0	0	6	24
	L-Lysine	4	0	0	0	0	0	0	7	28
	L-Valine	4	0	0	0	0	0	0	4	16
	L-Phenylalanine	4	0	0	0	0	0	0	4	16
	L-Threonine	4	0	0	0	0	0	0	4	16
	L-Tryptophan	4	0	0	0	0	0	0	1	4
	L-Methionine	4	0	0	0	0	0	0	3	12
	DL-Methionine	4	0.3	1	0.3	1	0.2	0.8	0	0
Total protein			42.5	170	42.5	170	85	340	85	340
Carbohydrate	Maltose Dextrin	4	115.2	461	25.6	102	16.4	65.6	16.4	65.6
Fat	Corn Oil	9	8.5	77	8.5	77	5.4	48.6	5.4	48.6
Fat	Oliver Oil	9	28.4	256	28.4	256	18.2	163.8	18.2	163.8
Fat	Safflower Oil	9	2.7	24	2.7	24	1.7	15.3	1.7	15.3
Fiber	Cellulose	0	10	0	10	0	6.4	0	6.4	0
Fiber	Xanthan Gum	0	3	0	3	0	1.9	0	1.9	0
Mineral	Mineral Mix #210011	0.5	8.8	4	8.8	4	5.6	2.6	5.6	2.6
Vitamin	Vitamin Mix # 310011	3.8	2.5	9	2.5	9	1.6	6.1	1.6	6.1
Vitamin	Choline Bitartrate	0	0.5	0	0.5	0	0.3	0	0.3	0
Ethanol	95% Ethanol	5.4	0	0	67ml	358	67ml	358	67ml	358
Total				1000		1000		1000		1000
NP, 17 kcal% normal-protein diet; HP, 34 kcal% high-protein diet; HP(no gln), 34 kcal% glutamine-deficient high-protein diet; AA, amino acid; EAA, essential amino acid.										

Table S2. Primer sequences used in the experiments

Name	Forward Primer	Reverse Primer
siRNA		
Si-NC	UUCUCCGAACGUGUCACGUTT	ACGUGACACGUUCGGAGAATT
Si-*Gls1#1*	GAUUUGCUGUUCUAUACAAUU	UUGUAUAGAACAGCAAAUCUU
Si-*Gls1#2*	GAGUGUAUGGAUAUGUUAAGA	UUAACAUAUCCAUACACUCUU
**Primer sequences for plasmids Construction**		
ΔPOLR2H-1	CCGGAATTCGCCACCATGGACTTGGGTGACAAGTTTCGGTT	CCGGAATTCGGATCCGAGCTCGGTACCAAGCTTAACTAGC
ΔPOLR2H-2	CCGGAATTCGGAGATGAAACTTCTACTGAAGCAG	CCGGAATTCTACAGGGTAAATTTGAATGTTTACATCTAAGATT
ΔPOLR2H-3	CCGGAATTCCTCGAGTCTAGAGGGCCCTTCTACCCATA	CCGGAATTCCTCAATCCTGTACACTTTTCCATACATTACAT
ΔPOLR2E-1	CCGGAATTCGCCACCATGCAGATGTTTGTGTTCTTTCCAG	CCGGAATTCGGATCCGAGCTCGGTACCAAGCTTAACTAGC
ΔPOLR2E-2	CCGGAATTCGAGCACGAGCTAGTCCCTGAGCACGT	CCGGAATTCGTCGGTGGGGTCATCGTTGTGGGC
ΔPOLR2E-3	CCGGAATTCCTCGAGTCTAGAGGGCCCTTCTACCCATA	CCGGAATTCCGTGATGTTGATGAGCAGCTCCTGCTG
ΔKGA-1	CTAGCTAGCGCCACCATGATTCCTGACTTTATGTCTTTT	CTAGCTAGCGGATCCGAGCTCGGTACCAAGCTTAACTA
ΔKGA-2	CTAGCTAGCTTCAGTAATGCAACGTTTCAGTCTGAAAGAG	CTAGCTAGCCACAAACTTTCTTCTAAATGCTTGTGTCAAC
ΔKGA-3	CTAGCTAGCAGACACTTTGCAAAAAAACTTGATCCTC	CTAGCTAGCTCCAACATATTCATTACCAGCCATCTTATTC
ΔKGA-4	CTAGCTAGCCTCGAGTCTAGAGGGCCCTTC	CTAGCTAGCCAAATTATCATAGTTATGGAAATTACACAGAGAAAC
GAC-S314A	CGATATGTTGGAAAAGAGCCGGCTGGACTAAGATTCAACAAACT	AGTTTGTTGAATCTTAGTCCAGCCGGCTCTTTTCCAACATATCG
KGA-S286A	GTTCCCTTCTGTCTTCAGGCCTGTGTAAAACCTTTGAAA	TTTCAAAGGTTTTACACAGGCCTGAAGACAGAAGGGAAC
KGA-ΔL	TCGGGGATGCTGCGGTCGCCCGCCGGCGTGAGC	CGACCGCAGCATCCCCGAGCCTCGCAGCCGCAT
**Primer sequences for qPCR**		
m-Acaca	ATGGGCGGAATGGTCTCTTTC	TGGGGACCTTGTCTTCATCAT
m-Acly	ACCCTTTCACTGGGGATCACA	GACAGGGATCAGGATTTCCTTG
m-Fasn	TGGGTTCTAGCCAGCAGAGT	TACCACCAGAGACCGTTATGC
m-β-actin	GGACTCCTATGTGGGTGACG	CTTCTCCATGTCGTCCCAGT

Table S3. Key Reagents used in the experiments

REAGENT or RESOURCE	SOURCE	IDENTIFIER
Antibodies		
Mouse monoclonal anti-DYKDDDDK Tag (9A3)	Cell Signaling Technology	Cat# 8146; RRID: AB_10950495
Mouse monoclonal anti-Myc-Tag (9B11)	Cell Signaling Technology	Cat# 2276; RRID: AB_331783
Rabbit monoclonal anti-HA tag	Cell Signaling Technology	Cat# 3724； RRID: AB_1549585
Rabbit polyclonal anti-KGA/GAC	Proteintech	Cat# 12855-1-AP, RRID: AB_2110381
Mouse monoclonal anti-POLR2E (C-10)	Santa Cruz Biotachnology	Cat# sc-390902, RRID: AB_3094666
Mouse monoclonal anti-Pol I/II/III RPB8 (B-2)	Santa Cruz Biotachnology	Cat# sc-398512, RRID: AB_3094667
Rabbit monoclonal anti-ACLY	Abclonal	Cat# A3719, RRID: AB_2863129
Rabbit monoclonal anti-Acetyl-CoA Carboxylase	Cell Signaling Technology	Cat# 3676, RRID: AB_2219397
Rabbit polyclonal anti-Fatty Acid Synthase	Cell Signaling Technology	Cat# 3189, RRID: AB_2100798
Rabbit monoclonal anti-ACTB	Abclonal	Cat# AC038, RRID: AB_2863784
Rabbit monoclonal anti-GAPDH	Cell Signaling Technology	Cat# 2118, RRID: AB_561053
Rabbit polyclonal anti-Histone H3	Cell Signaling Technology	Cat# 9715, RRID: AB_331563
Mouse Control anti-IgG	Abclonal	Cat# AC011; RRID: AB_2770414
Rabbit Control anti-IgG	Abclonal	Cat# AC005, RRID: AB_2771930
Rabbit monoclonal anti-GLS2	Cell Signaling Technology	Cat# 85934, RRID: AB_3090191
Rabbit polyclonal anti-GLUD1	Abclonal	Cat# A7631, RRID: AB_2768145
Rabbit polyclonal anti-GLUL	Abclonal	Cat# A5437, RRID: AB_2863503
Rabbit polyclonal anti-SLC1A5	Abclonal	Cat# A12676, RRID: AB_2759522
HRP-conjugated Affinipure Goat Anti-Rabbit IgG(H+L)	Proteintech	Cat# SA00001-2, RRID: AB_2722564
HRP-conjugated Affinipure Goat Anti-Mouse IgG(H+L)	Proteintech	Cat# SA00001-1, RRID: AB_2722565
Goat anti-Mouse IgG (H+L) Cross-Adsorbed Secondary Antibody, Alexa Fluor 488	Abcam	Cat# ab150117; RRID: AB_2688012
RNA pol II antibody	Active Motif	Cat# 39097, RRID:AB_2732926
Bacterial and virus strains		
E. coli: Stbl3	Shifengsw	Cat# C3040I
AAV8-TBG-GLS1	Genomeditech	This paper
AAV8-TBG-Vector	Genomeditech	This paper
AAV8-TBG-POLR2E WT	Genomeditech	This paper

AAV8-TBG-POLR2H WT	Genomeditech	This paper
AAV8-TBG-ΔPOLR2E-3	Genomeditech	This paper
AAV8-TBG-ΔPOLR2H-1	Genomeditech	This paper
AAV8-TBG-shNC	Genomeditech	This paper
AAV8-TBG-shGLS1	Genomeditech	This paper
Biological samples		
N/A	N/A	N/A
Chemicals, peptides, and recombinant proteins		
Bodipy	Thermo Fisher Scientific	Cat# D3922
Hoechst 33342	Thermo Fisher Scientific	Cat# 62249
DAPI	MCE	Cat# HY-D0814
Cycloheximide	MCE	Cat# HY-12320
MG132	MCE	Cat# HY-13259
Ethanol	Sinopharm Chemical Reagent Co.Ltd	Cat# 100092008
α-Amanitin	MCE	Cat# HY-19610
Glutamine	Sigma-Aldrich	Cat# G3126
Glutamate	Sigma-Aldrich	Cat# 49621
Arginine	MCE	Cat# HY-N0455
Histidine	MCE	Cat# HY-N0832
Tryptophan	MCE	Cat# HY-N0623
Threonine	MCE	Cat# HY-W012874
Phenylalanine	MCE	Cat# HY-N0215
Alanine	MCE	Cat# HY-N0215
Methionine	MCE	Cat# HY-13694
Isoleucine	MCE	Cat# HY-N0771
Serine	MCE	Cat# HY-N0650
Valine	MCE	Cat# HY-N0717
Asparagine	MCE	Cat# HY-N0667
Cysteine	MCE	Cat# HY-Y0337
Glycine	MCE	Cat# HY-Y0966
Tyrosine	MCE	Cat# HY-N0473
Leucine	MCE	Cat# HY-N0486
Lysine	MCE	Cat# HY-B2236
L-Proline	MCE	Cat# HY-Y0252
Aspartate	MCE	Cat# HY-N0667
Caisein	Sigma-Aldrich	Cat# C7078
Critical commercial assays		
Triglyceride kit	Fosun Diagnostics	Cat# 1.02.1803
HiScript III RT SuperMix for qPCR	Vazyme	Cat# R323-01
AceQ® Universal SYBR® qPCR Master Mix	Vazyme	Cat# Q511-02
Primer STAR HS DNA Polymerase	TaKaRa	Cat# R010b
Lipofectamine 3000 Transfection Reagent	Thermo Fisher Scientific	Cat# L3000001

Lipofectamine 2000 Transfection Reagent	Thermo Fisher Scientific	Cat# 11668019
Nuclear and Cytoplasmic Protein Extraction Kit	Beyotime	Cat# P0027
Steady-Lumi™ II Firefly Luciferase Assay Kit	Beyotime	Cat# RG059
CellTiter-Glo® Luminescent Cell Viability Assay	Promega	Cat# G7572
TRIzol reagent	TaKaRa	Cat# 9109
protein A/G agarose	Beyotime	Cat# P2055
SimpleChIP® Enzymatic Chromatin IP Kit	Cell Signaling Technology	Cat# 9003
BeyoClick™ EU RNA Synthesis Kit	Beyotime	Cat# R0301S
Experimental models: cell lines		
Human: HEK293T cells	Cell Bank/Stem Cell Bank, Chinese Academy of Sciences	Cat# CRL-3216™
Mouse: AML12 cells	ATCC	Cat# CRL-2254
Human: LO2 cells	ATCC	Cat# CRL-12461
Experimental models: Organisms/strains		
C57BL6/J wide type mice	Shanghai SLAC Laboratory Animal Co., Ltd.	N/A
Deposited data		
Liver tissue RNA sequencing data of mice	This paper	GSA: CRA014391
RNA sequencing data of healthy controls and patients with AH	GSEA	GEO: GSE28619
Liver tissue RNA sequencing data of control, ASH, or AH mice	GSEA	GEO: GSE97234
IP-MS data of oe-GLS1	This paper	PRIDE: PXD050603
ChIP-seq data of oe-GLS1	This paper	GSA: CRA027295
Recombinant DNA		
pcDNA3.1-Vector	YouBio	This paper
pcDNA3.1-GAC-Myc	YouBio	This paper
pcDNA3.1-KGA-Flag	YouBio	This paper
pcDNA3.1-POLR2H-HA	YouBio	This paper
pcDNA3.1-POLR2E-HA	YouBio	This paper
pcDNA3.1-∆KGA-1-Flag	This paper	This paper
pcDNA3.1-∆KGA-2-Flag	This paper	This paper
pcDNA3.1-∆KGA-3-Flag	This paper	This paper
pcDNA3.1-∆KGA-4-Flag	This paper	This paper
pcDNA3.1-∆POLR2E-1-HA	This paper	This paper
pcDNA3.1-∆POLR2E-2-HA	This paper	This paper
pcDNA3.1-∆POLR2E-3-HA	This paper	This paper
pcDNA3.1-∆POLR2H-1-HA	This paper	This paper

pcDNA3.1-ΔPOLR2H-2-HA	This paper	This paper
pcDNA3.1-ΔPOLR2H-3-HA	This paper	This paper
pcDNA3.1-GAC-S314A-Myc	This paper	This paper
pcDNA3.1-KGA-S286A-Flag	This paper	This paper
pcDNA3.1-KGA-ΔL-Flag	This paper	This paper
pGL3-Basic plasmid	YouBio	This paper
pGL3-Promoter plasmid	YouBio	This paper
Rodent Diets		
Lieber-DeCarli control liquid diet	Research Diets	Cat# 710027
Lieber-DeCarli ethanol liquid diet	Research Diets	Cat# 710260
Lieber-DeCarli high-protein ethanol liquid diet	Research Diets	Cat# 710262
Lieber-DeCarli high-protein ethanol liquid diet, no glutamine	Research Diets	Cat# Lieber-0422
Software and algorithms		
ImageJ	ImageJ	https://imagej.nih.gov/ij/
GraphPad Prism	GraphPad Prism	https://www.graphpad.com/
R studio	RStudio	https://www.r-project.org/
Image lab	Bio-Rad	https://www.bio-rad.com/
Biosystems	Azure	https://biosystems.global/
LAS X	Leica	https://www.leica-microsystems.com/
Adobe Illustrator	Adobe	https://www.adobe.com/products/illustrator.html
GSA database	GSA	https://ngdc.cncb.ac.cn/
Other		
Opti-MEM Medium	GIBCO	Cat# 31985070
HepatoZYME-SFM Medium	GIBCO	Cat# 17705021
Penicillin/Streptomycin	GIBCO	Cat#15140-122
DMEM/F12 Medium	GIBCO	Cat#11320033
HG-DMEM Medium	GIBCO	Cat#11965167
DMEM (deprived of glutamine) Medium	GIBCO	Cat#11054020
RPMI 1640 Medium	GIBCO	Cat# 31800022
LG-DMEM Medium	GIBCO	Cat# 31600034
RIPA	Beyotime	Cat# P0013B
Phenylmethanesulfonyl fluoride（PMSF）	Beyotime	Cat# ST507
4×Protein SDS PAGE Loading Buffer	Takara	Cat# 9173
G-250	Sangon	Cat# A100615-0050
Triton X-100	Beyotime	Cat# ST795
Fetal Bovine Serum (FBS)	GIBCO	Cat# 16000044
4% PFA	Ribiology	Cat# R1001
Cell lysis IP buffer	Beyotime	Cat# P0013
